# Characterisation of Genetic Variation in *ST8SIA2* and Its Interaction Region in NCAM1 in Patients with Bipolar Disorder

**DOI:** 10.1371/journal.pone.0092556

**Published:** 2014-03-20

**Authors:** Alex D. Shaw, Yash Tiwari, Warren Kaplan, Anna Heath, Philip B. Mitchell, Peter R. Schofield, Janice M. Fullerton

**Affiliations:** 1 Neuroscience Research Australia, Sydney, New South Wales, Australia; 2 Schizophrenia Research Institute, Sydney, New South Wales, Australia; 3 School of Medical Sciences, Faculty of Medicine, University of New South Wales, Sydney, New South Wales, Australia; 4 Peter Wills Bioinformatic Centre, Garvan Institute, Sydney, New South Wales, Australia; 5 School of Psychiatry, University of New South Wales, Sydney, New South Wales, Australia; 6 Black Dog Institute, Sydney, New South Wales, Australia; Seoul National University College of Medicine, Republic of Seoul Korea

## Abstract

Alpha-2,8-sialyltransferase 2 (ST8SIA2) is an enzyme responsible for the transfer of polysialic acid (PSA) to glycoproteins, principally the neuronal cell adhesion molecule (NCAM1), and is involved in neuronal plasticity. Variants within *ST8SIA2* have previously shown association with bipolar disorder, schizophrenia and autism. In addition, altered PSA-NCAM expression in brains of patients with schizophrenia or bipolar disorder indicates a functional dysregulation of glycosylation in mental illness. To explore the role of sequence variation affecting PSA-NCAM formation, we conducted a targeted re-sequencing study of a ∼100 kb region – including the entire ST8SIA2 gene and its region of interaction with NCAM1 – in 48 Caucasian cases with bipolar disorder using the Roche 454 platform. We identified over 400 DNA variants, including 47 putative novel variants not described in dbSNP. Validation of a subset of variants via Sequenom showed high reliability of Roche 454 genotype calls (97% genotype concordance, with 80% of novel variants independently verified). We did not observe major loss-of-function mutations that would affect PSA-NCAM formation, either by ablating ST8SIA2 function or by affecting the ability of NCAM1 to be glycosylated. However, we identified 13 SNPs in the UTRs of *ST8SIA2*, a synonymous coding SNP in exon 5 (rs2305561, P207P) and many additional non-coding variants that may influence splicing or regulation of *ST8SIA2* expression. We calculated nucleotide diversity within *ST8SIA2* on specific haplotypes, finding that the diversity on the specific “risk” and “protective” haplotypes was lower than other non-disease-associated haplotypes, suggesting that putative functional variation may have arisen on a spectrum of haplotypes. We have identified common and novel variants (rs11074064, rs722645, 15∶92961050) that exist on a spectrum of haplotypes, yet are plausible candidates for conferring the effect of risk and protective haplotypes via multiple enhancer elements. A Galaxy workflow/pipeline for sequence analysis used herein is available at: https://main.g2.bx.psu.edu/u/a-shaw-neura/p/next-generation-resources.

## Introduction

Polysialic acid (PSA) is an important carbohydrate that plays a crucial role in neural development and plasticity [1]–[3] (reviewed in [4], [5]). Homopolymers of α2,8-linked sialic acid are synthesised in the Golgi by sialyltransferase enzymes, of which there are six in the human genome. Two sialyltransferases, encoded by *ST8SIA2* on human chromosome 15q26.1 and *ST8SIA4* on 5q21.1, catalyse the transfer of long chains (>50 residues) of PSA onto their major substrate, neuronal cell adhesion molecule 1 (NCAM1) [2], [6] (reviewed in [5]), to form PSA-NCAM. Expression of PSA-NCAM is particularly high in early brain development [1]. PSA addition to NCAM1 increases the hydrated volume of cells and limits the adhesive properties of NCAM1, enabling neuronal migration, dendrite formation, axon targeting and synaptic plasticity (reviewed in [4]). Mice expressing only PSA-free NCAM after knockout of the relevant sialyltransferase genes (*st8sia2* ^−/−^; *st8sia4* ^−/−^), show severe malformation of major brain axon tracts, which are reminiscent of those observed in patients with schizophrenia [7], [8]. Alterations in the expression of PSA-NCAM in the adult post-mortem brain – particularly in the amygdala and hippocampal dentate gyrus & CA1 regions – have been shown in patients with schizophrenia, bipolar disorder, major depression and drug-refractory temporal lobe epilepsy [9]–[12]. Further, expression of PSA-NCAM is modulated in rats by treatment with common antipsychotics [13]–[15].

There is growing genetic evidence of the involvement of *ST8SIA2* (also known as *SIAT8B* or *STX*) in conferring risk to mental illness. We and others have previously identified the 15q25–26 genomic region as harbouring a mental illness risk gene through linkage in family cohorts with bipolar spectrum disorder [16], bipolar disorder and schizophrenia [17], [18] or psychosis [19]. We subsequently found evidence of disease association with variants in *ST8SIA2* in both bipolar disorder and schizophrenia [20]. Other groups have shown association with schizophrenia in Japanese and Chinese cohorts in the promoter region of *ST8SIA2* [21], [22], which was selected as a candidate gene after *NCAM1* was implicated in disease risk [23]. More recently, genome-wide association studies have implicated SNPs downstream of *ST8SIA2* in risk of bipolar disorder [24], and variants in intron 2 have also been associated with autism spectrum disorder (verbal subtype) [25]. Furthermore, a 520 kb hemizygous deletion of three genes including *ST8SIA2* has been identified in a patient with autism spectrum disorder [26]. Hence, characterisation of the nucleotide variation in *ST8SIA2* in patients with a variety of mental illnesses is an important step in understanding the molecular mechanisms through which this gene increases disease risk.

Recent advances in high-throughput sequencing have allowed the rapid sequencing of whole gene loci in multiple individuals [27]. We have exploited this technique to sequence the coding, intronic and flanking sequence of *ST8SIA2* and its region of interaction in *NCAM1*, in lymphocyte DNA from individuals with bipolar disorder. We incorporated a number of methods into our analysis pipeline for quality control in variant detection and base-calling. Altogether, we have identified 396 single nucleotide variants including 42 novel single nucleotide variants across 95 kb of genomic DNA in *ST8SIA2*, and 21 variants, 5 of which are novel, in a 6 kb region of *NCAM1*. We utilised data from the 1000 Genomes Project [28] to identify variants unique to bipolar individuals, and those which are at altered frequency in bipolar cases compared to the 1000 Genomes population panels [29] with the same racial background. We examined the haplotypic background of each variant in *ST8SIA2* with respect to a previously identified risk haplotype [20] and explored evidence of functional impact by comparisons with ENCODE data [30], [31] and co-localisation with *ST8SIA2*-specific enhancer elements that contribute to altered target gene expression [32]. Through an integrative analysis of the above methods, we have identified and verified the most compelling candidate variants for increasing susceptibility to mental illness by modulating ST8SIA2 function.

## Results

### 454 Sequencing and Mapping

**Figure 1:**
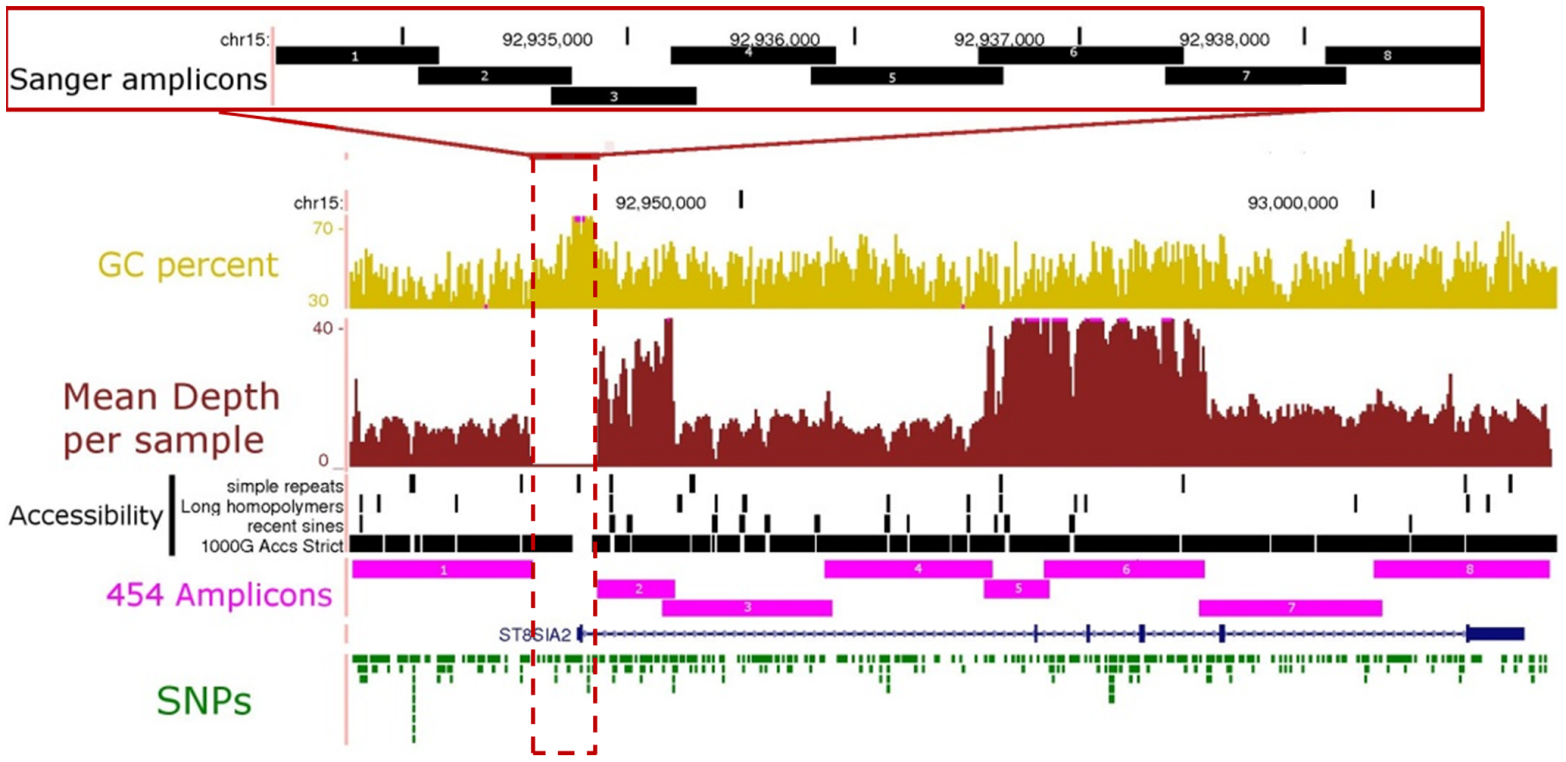
Coverage of *ST8SIA2* gene locus by 454 and Sanger sequencing. The 96∼20 kb flanking sequence (chr15∶92,919,255–93,013,920 bp; UCSC Genome Browser hg19 http://genome.ucsc.edu) was divided into eight long-range PCR amplicons (454 Amplicons 1–8, in pink) for library preparation prior to 454 sequencing. The mean depth of coverage across all 48 samples for each long-range amplicon is shown in burgundy. A GC-rich region surrounding the promoter and exon 1 was sequenced via Sanger sequencing using 8 short overlapping amplicons (Sanger amplicons 1–8; inset). Pink dots on the GC percent or mean depth per sample tracks indicate loci where the level of GC richness, or read depth is above the range indicated. Regions which failed to amplify, or which contained simple repeats, long homopolymers (length > = 10), or short interspersed elements (SINEs) tended to have lower depth of coverage, and were inaccessible to next-generation sequencing in phase I of the 1000 genomes project (1000 G Accs strict).

**Figure 2:**
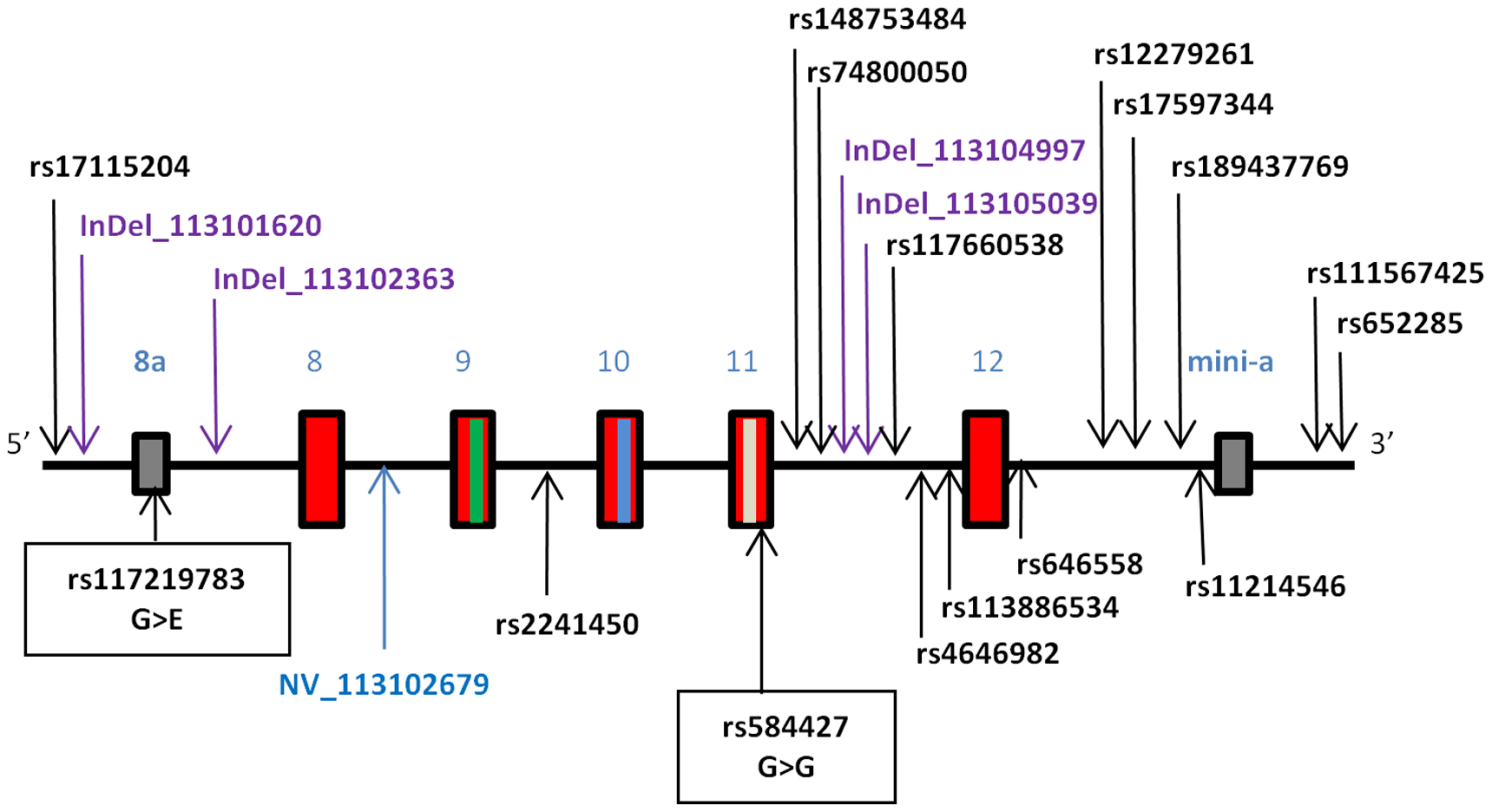
Region of *NCAM1* gene covered via Roche 454 sequencing. The sequenced region spanned 6.6(chr11∶113,100,972–113,107,571 bp; hg19) and included exons 8–12 (red boxes) and less frequently used exons 8a and mini-a (grey boxes) [52]. The locations of the 5^th^ and 6^th^ glycosylation sites and acidic patch required for glycosylation are in exons 9, 10 and 11 and are indicated by green, blue and white lines, respectively. The locations of SNPs identified in this study are indicated with arrows. Names of known SNPs are in black text, and novel SNPs are listed as NV (novel base substitutions) or InDel (novel insertion-deletion change) followed by their base position (hg19). Two coding SNPs located in exons 8a and 11 are marked with closed boxes, with their effect on amino acid sequence shown.

To characterise genetic variation that may quantitatively impact formation of PSA-NCAM, we performed massively parallel sequencing on the 454 GS-FLX platform of two regions that we hypothesise are associated with ST8SIA2-related developmental pathology in mental illness: 1) a ∼95 kb region including the entire coding and intronic regions and ∼20 kb of flanking sequence of *ST8SIA2* (hg19/GRCh37: chr15∶92,919,255–93,013,920 bp; Figure 1); and 2) a ∼6 kb region within *NCAM1* (hg19/GRCh37: chr11∶113,100,972–113,107,571 bp; Figure 2). This latter region contains the 5^th^ immunoglobulin-like domain and fibronectin-1 domain, and specifically the 5^th^ and 6^th^ glycosylation sites and acidic patch (that are critical for enzyme recognition, docking and polysialylation by ST8SIA2 to form PSA-NCAM [33]–[35].

We applied BWA mapping [36] to the entire genome to ensure that mapped reads were specific to regions amplified by long-range PCR, and to determine rates of target enrichment and non-specific amplification. We found that 96% of reads across all individuals mapped to a unique site within the human genome, and that 93% of reads that mapped to the human genome mapped within the target regions. The remaining mapping is likely due to some amplification of non-targeted regions by the long-range PCR primers.

### Coverage

Depth and uniformity of coverage are critical for sensitive SNP detection and accurate genotype calls, and are important considerations in assessing of the completeness of variant detection in targeted resequencing. Hence, we examined the mean depth of coverage across each base-pair of the *ST8SIA2* region (Figure 1). We found that depth of coverage dropped substantially and locally in regions of low complexity, which included simple repeat sequences and very long homopolymers (length ≥10). These regions are known to be difficult to base call accurately due to the deficiencies in sequencing chemistry of 454 [37]. Indeed, many of these regions were found to be less accessible to sequencing according to the “strict” stringency assessment of the human genome conducted in phase I of the 1000 Genomes Project [28]. Many of these regions also coincided with the ends of primate-specific (recently evolved) Alu repeat elements, which are rich in A and T homopolymers [38].

**Figure 3:**
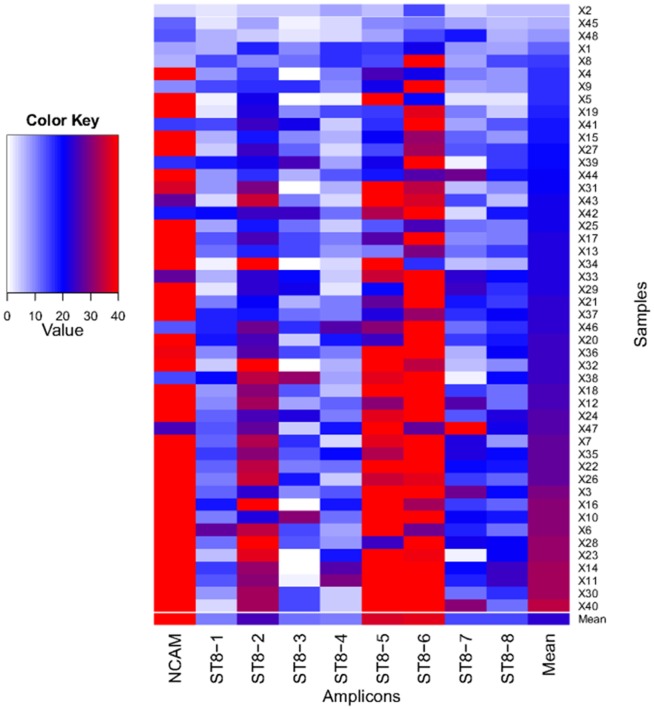
Heatmap of mean depth of coverage per amplicon and per sample. Amplicons are listed along the x-axis, and samples (x1-48) are listed along the y-axis. Depth of coverage is indicated according to the colour key, with white indicating <2 reads coverage and red indicating >35 reads coverage. The plot was created using R [63].

Aside from local sequence context, a major determinant of mean depth of coverage was inclusion within a specific long-range PCR amplicon, with the *NCAM* amplicon and *ST8SIA2* amplicons 2, 5 and 6 having the highest mean coverage (Figure 3). We assessed the depth of coverage in each sample, and also found variation between samples (Figure 3). These differences demonstrate that amplicon pooling and sample pooling steps each contributed to variation in the depth of sequence coverage within genomic regions. Overall, although some samples contained amplicons with poor coverage (Figure 3), the mean coverage for each amplicon across all samples was >10, a level sufficient for ascertainment of diploid genotypes.

### SNP calling

We used two different algorithms to call SNPs from 454-generated data: i) GS Reference Mapper (Refmapper), which is a vendor-supplied algorithm specific to the 454 platform, and calls SNPs based on alignment data from each individual independently; and ii) GATK [39], [40], which is not platform specific, and calls SNPs based on alignment data from all individuals combined. As raw GATK SNP calls require some level of post-hoc filtering, we initially used the GATK-recommended filtering criteria for whole genome SNP detection, and then increased the stringency to remove a subset of likely spurious variant calls (further details are supplied in Note S1; Figure S1). In order to maximise our ability to detect true SNPs for functional assessment, the union of both Refmapper and GATK algorithms (i.e., those SNPs that were called by at least one algorithm) formed our list of putative SNPs.

We compared the locus of SNPs called using each algorithm and found that 89% of called SNPs were common to both algorithms (Table 1). Taking only the subset of putative novel SNPs in each set, we found that each algorithm alone and the intersection between the algorithms generated approximately the same proportion of putative novel variants (8–9%), and ∼60% of the putative novel variants were called in both algorithms (Table 1). Twenty one SNPs filtered out in GATK were called as genuine variants by Refmapper. A further 23 SNPs were identified by only one algorithm.

**Table 1:** Comparison of SNP detection methods from 454 data, and identification of bona fide SNPs for functional analysis.

Set		n
Refmapper	All	383
	Novel	34 (9%)
GATK (passed filter)	All	349
	Novel	28 (8%)
GATK (failed filter)	All	232
Intersection	All	344
	Novel	24 (7%)
Union	All	388
	Novel	38 (10%)
Sanger	All	24
	Novel	5

The number of SNPs (n) which were detected individually by Refmapper and GATK are shown. The SNPs that were called by both algorithms (and passed filtering) are given in the intersection, and the union represents SNPs that were called by at least one package. SNPs detected in the 5.3 kb region sequenced via Sanger sequencing are also listed.

We sequenced a GC-rich region that was not amplified by long-range PCR using bi-directional Sanger sequencing (Figure 1). SNPs were called using Consed and the Phred/Phrap/Polyphred software suite [41], [42], with manual inspection of chromatograms for SNP identification and genotyping. Non-reference SNP and genotype calls were only made if the non-reference base was called in both the forward and reverse reads. Using this methodology we identified 24 additional SNPs in the *ST8SIA2* locus (19 known, 5 novel).

### Genotype calling and accuracy

Diploid genotype calling from high throughput sequencing relies on a minimal level of read coverage in each individual at each base position to accurately distinguish true heterozgous sites from sites with random base-calling errors. Due to variation in coverage across regions and across amplicons (Figure 3), we reasoned that a proportion of the targeted region in each individual was marginal, i.e. was covered to a depth below that required for statistically confident heterozygote calling (coverage <10). In order to use data from these regions, and other regions with low local coverage, we used the probabilistic genotype calling module within GATK. This module uses read data at all sites, even those with low coverage, in the simultaneous estimation of cohort allele frequency and calculation of genotype likelihoods. The diploid genotype with the highest likelihood is assigned as the genotype call, along with a likelihood ratio calculation of the confidence of this call [denoted Genotype Quality (GQ)].

In order to assess the overall accuracy of applying this mode of genotype calling to our 454 sequence data, we compared the GATK 454-derived genotypes with genotypes determined directly via Illumina genotyping assay for the same individuals from an earlier study [20], [43], which were assumed to be 100% accurate. The available Illumina data contained 2133 genotype calls from 50 SNPs (minor allele frequency [MAF] average 0.29; range 0.01–0.49), including 1081 (50.7%) non-homozygous-reference calls. One sample (# 10) was excluded from further analysis based on high genotype discordance between platforms (∼50%). The overall genotype discordance across the remaining individuals was low (∼2%; Table 2). We reasoned that some genotype calls may have been made at low confidence in regions with low coverage, and that these could be removed from all genotype calls to reduce discordance. Indeed we found that eliminating low confidence genotype calls (GQ <10), which are most subject to random error, reduced the discordance 2-fold at the expense of a decrease in the number of genotypes called (Table 2).

**Table 2:** Genotype concordance between 454-generated genotype calls and those directly genotyped via Illumina GoldenGate or Illumina 660 W.

Set	Call accuracy	Call rate	Het call accuracy	Het call rate
All	0.98	0.95	0.95	0.93
Low confidence removed	0.99	0.89	0.98	0.89

Genotype calls are divided into two categories: all calls; or confident genotype calls based on Genotype Quality (GQ) ≥ 10. Call accuracy gives the concordance between the SNP genotype derived from 454 data and Illumina-generated data across all samples excluding missing data, and the call rate incorporates missing data on an individual basis. These are calculated for all genotypes, and heterozygous (het) calls only.

Genotype calling for Sanger data was performed using Polyphred. Only two SNPs were available with known genotypes from the Illumina genotyping data for assessing the accuracy of Sanger genotype calling. We found that all known genotypes were accurately called in 44 individuals with available known genotypes (88 known genotypes, 14 non-reference).

### Comparison to 1000 Genomes Project data

Having ascertained a list of putative SNPs that were observed in Caucasian individuals with bipolar disorder, we were able to assess our ability to detect SNPs present in the general population by comparing our detected variants to those of Caucasian Europeans represented in phase I of the 1000 Genomes (1 kG) project (CEU and GBR populations; n = 174).

**Figure 4:**
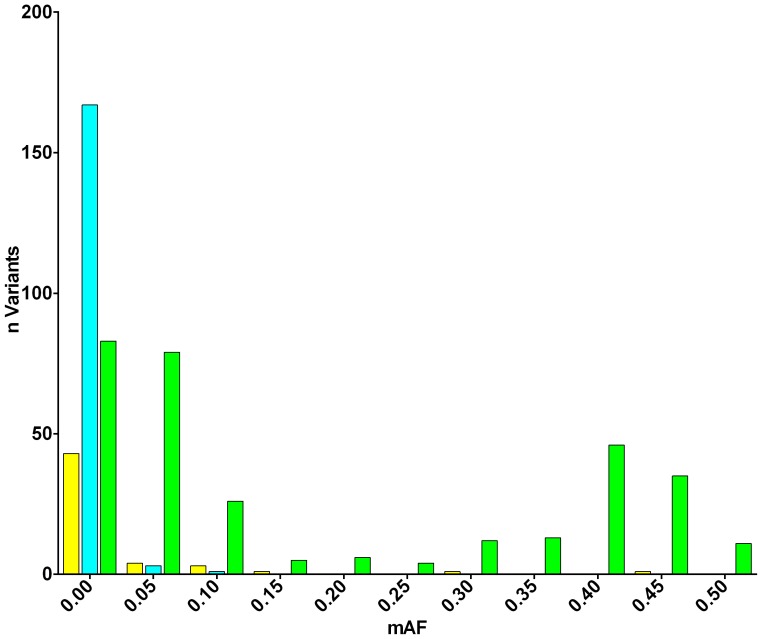
Comparison of detected SNP variants with all SNPs detected in 1 kG data. The minor allele frequency (MAF) of all SNPs identified in the sequenced region from 174 Caucasian Europeans from phase I of the 1 kG project (n = 519) and 48 bipolar disorder cases (n = 412) were compared. SNPs found in bipolar individuals only (yellow) and 1 kG individuals only (light blue) were predominantly rare alleles whereas those found in both sets (green) were predominantly common.

We found that many rare SNPs (MAF <0.05) were found to be unique to either the bipolar cohort or 1 kG individuals (Figure 4), which is expected due to the inevitable sampling bias which occurs when sampling small numbers of subjects from a population. In contrast, 100% of common variants (MAF ≥0.1) found in the 1 kG cohort were also found in our bipolar cohort, confirming our ability to detect common variants in the population. A small number of common variants found in our cohort (n = 3) were apparently not detected in 1 kG: however, manual inspection showed that these SNPs were adjacent to indels, or low-complexity sequence, and therefore were probably filtered from the list of integrated variants in the 1 kG cohort. We excluded these putative SNPs from further analysis.

To determine which variants might be associated with illness, we next compared the allele frequencies of all SNPs detected in *ST8SIA2* and *NCAM1* in the 48 bipolar cases with those reported in the 174 1 kG individuals. While the 1 kG population may contain individuals with bipolar disorder, this would be expected to be at the population rate of around 1.5%, so it can be treated as an unscreened control population. We found that two SNPs in *NCAM1* (rs584427 and rs646558, representing ∼10% of all observed) and 53 SNPs in *ST8SIA2* (∼14% of all observed) showed a frequency difference of 5% or more between the two populations (Table 3, Table S1).

**Table 3:** Summary of genetic variation identified in NCAM1.

SNP	Chr11 Position	poly^a^	Freq (BP)	Freq (1 kG)	FreqDIFF
rs17115204	113101143	T/C	0.011	0.006	0.005
11∶113101620	113101620	-/T	0.030	0.000	0.030#
**Exon 8a**					
rs117219783*	113101958	A/G	0.021	0.012	0.010
11∶113102363 v	113102363	-/C	0.010	0.000	0.010
**Exon 8**					
11∶113102679	113102679	G/C	0.010	0.000	0.010
**Exon 9**					
rs2241450	113103259	T/C	0.011	0.006	0.005
**Exon 10**					
**Exon 11**					
rs584427* v	**113103996**	**T/G**	**0.340**	**0.450**	**−0.110#**
rs148753484	113104198	-/CACCCCAA	0.110	0.088	0.022
rs74800050	113104456	T/C	0.032	0.018	0.014
11∶113104997	113104997	-/T	0.020	0.000	0.020#
11∶113105039	113105039	-/T	0.010	0.000	0.010
rs117660538	113105158	G/C	0.011	0.012	−0.001
rs4646982 v	113105405	G/T	0.319	0.300	0.019
rs113886534	113105410	G/A	0.021	0.012	0.010
**Exon 12**					
**rs646558 v**	**113105907**	**A/C**	**0.245**	**0.194**	**0.051**
rs12279261	113106455	G/A	0.117	0.159	−0.042
rs17597344	113106464	A/G	0.043	0.065	−0.022
rs189437769	113106527	T/C	0.011	0.012	−0.001
rs11214546	113106528	T/G	0.043	0.053	−0.010
**Mini-exon a**					
rs111567425	113107077	A/G	0.011	0.035	−0.025
rs652285	113107204	A/G	0.096	0.053	0.043

The location of each variant (NCBI 37/hg19 build) relative to known exons is shown, with exon locations indicated, and coding SNPs indicated with asterisks. The minor allele frequency (MAF) of each variant in all samples (BP+1 kG) and the BP and 1 kG cohorts separately is given. The frequency difference (freqDIFF) was calculated relative to 1 kG allele frequency, with absolute frequency differences greater than 5% indicated in bold text, and those with p values <0.1 indicated with an asterisk.

a For each polymorphism, the minor allele is listed first.

v SNPs verified by direct genotyping are indicated.

### Nucleotide diversity on specific ST8SIA2 haplotype backgrounds

We previously identified specific haplotypes which were associated with increased or decreased disease risk, and were defined by six SNPs within the *ST8SIA2* locus [20]. In order to identify potentially functional variants which lie specifically on these haplotypes, we performed haplotype phasing and imputation using the BEAGLE algorithm. The data used as input was combined from Illumina genotypes, Sanger genotypes and 454 genotype likelihoods from GATK (excluding data in regions with potential allelic bias; see Note S2). Genotypes from 1 kG phase I Caucasian European individuals (n = 174) were used as a reference panel.

**Figure 5:**
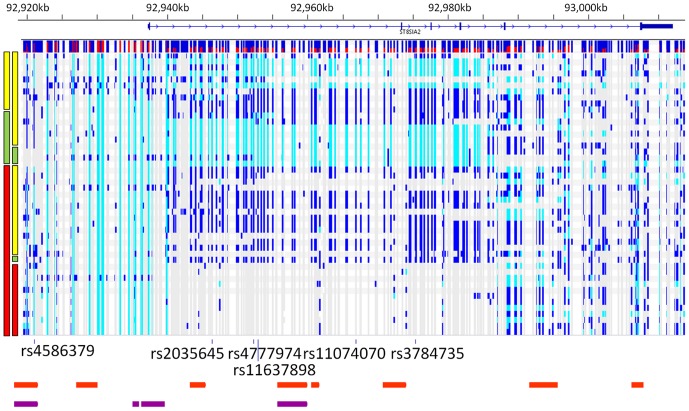
Haplotype phasing of all detected nucleotide variants in *ST8SIA2* gene region. The gene structure is shown above, with the minor allele frequency of each SNP detected shown by the histogram immediately below the gene (red represents frequency of alternate allele). The red, green and yellow bars on the left indicate the haplotype on which the detected variation resides (risk  =  red; protective  =  green; other haplotypes  =  yellow). Detected variation with respect to the specific haplotype on which it resides is shown to the right of haplotype bars, where non-reference alleles are coloured in light blue (homozygous) and dark blue (heterozygous). The locations of the six SNP markers that were used to define the haplotypes are shown below the variation. The location of the PreSTIGE enhancer elements within the 454 sequenced region from human skeletal muscle myoblasts (HSMM) are indicated in orange, and from neuronal precursor cells (NPC) are indicated in purple. PreSTIGE enhancer locations were obtained from http://genetics.case.edu/prestige/).

After phasing and imputation, 40 bipolar chromosomes were predicted to have the risk haplotype, whereas 13 bipolar chromosomes were predicted to have the protective haplotype (Figure 5). The remaining 41 bipolar chromosomes harboured neither risk nor protective haplotypes, and were designated as “other”. A large number of common variants in the bipolar individuals appear to have arisen through co-segregation of alleles in a large haplotype block between the 1st and 5th exons of *ST8SIA2* (Figure 5).

The nucleotide diversity across the sequenced region, as measured under the assumptions of an infinite site neutral allele model, was similar across *ST8SIA2* (θ = 5.42×10^−4^) to rates previously reported for other genes [44]. Surprisingly, the nucleotide diversity was lower on the designated risk and protective haplotypes (θ = 3.45×10^−4^ and 3.74×10^−4^) compared to other haplotypes (θ = 8.35×10^−4^). This may be indicative of allelic heterogeneity, i.e., that putative functional variants may have arisen on a spectrum of haplotypes, rather than restricted specifically to the risk haplotype previously associated with illness.

### Analysis of putative function

Having ascertained the location, diploid genotype and phase of identified SNPs, we turned our attention to the predicted functional impact of SNPs in the *ST8SIA2* and *NCAM1* sequenced regions, looking at the coding, transcribed and non-transcribed regions.

We found no non-synonymous SNPs in coding regions of *ST8SIA2*, although we identified a previously known synonymous SNP in exon 5 (rs2305561, P207P). We also identified a known SNP 5 bp from the 3′ end of exon 5 (rs11637874), for which the minor T allele was predicted to improve splice donor site efficiency (BDGP score: T allele  = 0.95; C allele  = 0.81). Allele frequencies for these two SNPs were similar to those in the 1 kG cohort (Table S1).

In *NCAM1*, we found a single non-synonymous SNP in exon 8a (rs117219783, G/A, G>E; Figure 2), which resulted in a non-conservative amino acid change (non-polar glycine to polar glutamic acid), although the MAF was not different to that observed in 1 kG data (MAF_(A)_ = 0.02 vs 0.01; χ^2^ = 0.37, *p* = 0.54; Table 3). This exon is rarely used, and was reported to exist in mRNA from testis (AK314589). We also observed a synonymous variant in exon 11 of *NCAM1* (rs584427, G/T, V540V), which showed some frequency distortion in the bipolar cases compared to 1 kG individuals (MAF_(T)_ = 0.34 vs 0.45 respectively; χ^2^ = 3.16, *p* = 0.08; Table 3). Two novel insertion/deletion SNPs (at 113,101,620 and 113,104,997 bp) showed nominal allelic association (χ^2^ = 5.37; *p* = 0.02 and χ^2^ = 3.57; *p* = 0.05, respectively). No variants were observed in the glycosylation regions or the acidic patch that would affect the ability of NCAM1 to be glycosylated.

The recent release of comprehensive functional genomics data [31] allowed us to assess the potential gene-regulatory impact of the non-coding variants we identified in *ST8SIA2*. To identify SNPs that may affect transcriptional regulation of *ST8SIA2* we looked at the localisation of SNPs within DNAse hypersensitivity sites (DHSs) in human Embryonic Stem Cells (hESC) or neuronal cell lines, surveyed in the ENCODE project [31], [45]. DHSs indicate a region of open chromatin, and DHS peaks (DHSPs) within the DNAse-Seq signal indicate chromatin that is potentially accessible for transcription factor binding, which could be modulated by sequence variants.

We found that 20 SNPs in total (five novel) were within DHSPs, five SNPs were within DHSPs in both hESC and neuronal cell lines (one novel), six within neuronal cell lines only (two novel) and nine within hESC cell lines only (two novel; Table 4). Consistent with enrichment for functional variants, assessment of Genomic Evolutionary Rate Profiling (GERP) scores revealed that 20% of DHSP-overlapping sites were conserved (GERP >2) compared to only 3% of non-DHS-overlapping sites. The five novel SNPs which were coincident with DHSPs (at 92937845, 92938435, 92962229, 92973414, 92984673) were present in only a single sequenced individual each. Two of these SNPs were conserved with a high GERP score (4.12 at 92937845 and 5.55 at 92973414) and one SNP was predicted to introduce a splice acceptor site 69 bp downstream of exon 2 (92973414, BDGP score  = 0.98 with A allele). Despite the conservation of a high proportion of DHSP-overlapping SNPs, the majority (n = 9) of conserved SNPs were neither within DHS nor transcribed. This indicates that either their transcriptional regulatory function is not captured in the cell lines surveyed, or that they impart a conserved function through another mode of regulation. A further survey of DHS clusters across 125 additional cell lines included in the ENCODE project revealed that the majority (n = 6) were not present in DHSs of any other cell lines (data not shown), suggesting that for these SNPs an alternate mode of regulation is responsible rather than a conserved transcriptional regulatory function in another cell type.

**Table 4:** Summary of transcribed and DHSP overlapping genetic variation identified in *ST8SIA2*.

SNP	Chr 15 position novel		mA Excl. to RISK	mA Excl. to PROT	Set	DHSP	GERP	poly^a^	Freq (BP)	Freq (1 kG)	freqDIFF
rs117156502	92926966		2	2	1	1,2		T/C	0.022	0.009	0.014
rs28496195	92926968		1	0	1	1,2		A/G	0.011	0.035	−0.023
**rs16946843**	**92928587**		**ND**	**ND**	**3a**	**2**		**T/C**	**ND**	**0.101**	**ND**
rs186699149	92935000		ND	ND	4	2,3			0.01	0	0.01
rs79668601	92936584		0	0	4	3		A/G	0.056	0.063	−0.008
rs138393846	92936774		ND	ND	4	2,3	4.28		0.01	0	0.01
**rs142883061**	**92937137**		**0**	**1**	**4**	**1**	−2.84	**C/T**	**0.053**	**0.106**	**−0.053**
**5′UTR&exon1**											
rs3743365*	92937268		0	0	4		1.08	G/T	0.1064	0.1523	−0.0459
rs3743364*	92937276		0	0	4		1.84	C/T	0.1064	0.1523	−0.0459
rs192224395	92937836		ND	ND	4	1,3	2.82		0.01	0	0.01
15∶92937845	92937845	Y	2	2	4	1,3	4.12	A/G	0.011	0	0.011
**rs3784745**	**92937858**		**0**	**0**	**4**	**1,2,3**	−0.45	**G/C**	**0.0851**	**0.1437**	**−0.0586**
**rs3784744** v	**92937884**		**0**	**1**	**4**	**1,2,3**	1.02	**C/G**	**0.053**	**0.106**	**−0.053**
15∶92938435	92938435	Y	ND	ND	4	2		A/G	0.011	0	0.011
rs73543829	92938826		0	0	1	3		C/T	0.011	0	0.011
rs11637377	92939998		0	1	1	3		T/C	0.022	0.055	−0.032
rs11857396	92940122		0	1	1	3		A/G	0.389	0.379	0.01
**rs722645** v	**92943323**		**0**	**1**	**1**	**3**		**G/A**	**0.489**	**0.563**	**−0.074**
rs28680784	92943349		0	1	1	3		T/C	0.424	0.391	0.033
**rs921846** v	**92943966**		**1**	**0**	**1**	**2**		**A/G**	**0.489**	**0.555**	**−0.066**
rs11074064^v^	92944662		0	1	1	3	3.91	A/G	0.457	0.471	−0.015
**rs11074065**	**92944835**		**0**	**1**	**1**	**3**		**A/G**	**0.371**	**0.48**	**−0.109#**
rs11853083	92951142		0	1	1	3	0.15	G/A	0.436	0.411	0.025
rs17599821	92951358		0	1	1	2,3		T/C	0.41	0.408	0.002
rs118032995	92952834		0	0	1	3		T/A	0.013	0.006	0.007
rs11637898	92952850		0	1	1	3		A/G	0.438	0.434	0.004
15∶92962229	92962229	Y	0	0	3a	1,2		C/T	ND	0	ND
15∶92969392	92969392	Y	ND	ND	3b	3			ND	0	ND
**Exon2**											
15∶92973414^v^	92973414	Y	2	2	1	1	5.55	A/G	0.011	0	0.011
**Exon3&4**											
rs112804054	92981903		2	2	1	1		C/A	0.011	0.009	0.002
15∶92984673	92984673	Y	0	0	1	2		A/G	0.011	0	0.011
**Exon5**											
rs2305561*	92987938		0	0	1		−0.19	G/C	0.1	0.115	−0.015
rs59585859	92998684		ND	ND	3b	3			ND	0.135	ND
**rs76405928**	**93000441**		**0**	**0**	**1**	**3**		**T/A**	**0.457**	**0.379**	**0.077#**
rs79846035	93000995		1	0	1	3	−2.22	A/G	0.054	0.049	0.005
rs74895969	93001035		0	1	1	3		A/G	0.043	0.055	−0.011
rs4777988	93002444		0	0	1	2		G/A	0.424	0.431	−0.007
**Exon6&3′UTR**											
rs139149207*^v^	93007785		0	0	1		5.45	A/C	0.011	0.003	0.008
rs2290492* v	**93007974**		**0**	**0**	**1**		4.64	**A/G**	**0.278**	**0.221**	**0.057**
rs12904773*	93008168		0	1	1			G/C	0.065	0.037	0.028
rs8035760* v	**93008472**		**0**	**0**	**1**			**A/T**	**0.359**	**0.19**	**0.169#**
rs1869774* v	**93010177**		**0**	**0**	**1**	**2**		**T/C**	**0.315**	**0.233**	**0.082#**
rs115781738*	93010192		2	2	1	2		C/G	0.011	0	0.011
rs116928729*	93010321		0	0	1			A/G	0.022	0.017	0.005
rs17600420*	93011135		0	0	1			G/A	0.304	0.345	−0.041
rs117763930*	93011237		0	0	1			A/G	0.022	0.017	0.005
rs145948851*	93011468		2	2	1			C/T	0.011	0	0.011
**rs11853992** v	**93012351**		**0**	**0**	**1**	**3**	2.51	**G/A**	**0.33**	**0.2787**	**0.0513**
rs147920939	93012426		0	0	1	3	−1.12	A/G	0.011	0.0086	0.0024

The location of transcribed and DHSP overlapping variants on chromosome 15 (hg19 build), with transcribed SNPs indicated with asterisks. SNP alleles (mA) identified exclusively on the risk haplotype are shown (0 =  not exclusive, 1 =  present on risk and other haplotypes, 2 =  present on risk haplotype only, ND  =  not determined). SNPs identified exclusively on the protective haplotype are shown (0 =  not exclusive, 1 =  present on protective and other haplotypes, 2 =  present on protective haplotype only, ND = not determined). The set from which the SNP was observed is given (1 =  GATK & Refmapper; 2 =  GATK only; 3a  =  Refmapper only; 3b  =  Refmapper only & filtered in GATK; 4 =  Sanger). Co-localisation with DNase I hypersensitivity site (DHS) peaks (neuronal = 1; hESC = 2; foetal brain = 3) are given. Genomic Evolutionary Rate Profiling (GERP) scores are provided for each variant that is within a GERP-conserved element. The nature of the polymorphism in each cohort is given (with minor allele listed first). The minor allele frequency (MAF) of each variant in the 47 bipolar cases and 174 Caucasian individuals (CEU and GBR) from the 1000 Genomes Project (1 kG) are shown separately. The frequency difference (freqDIFF) was calculated relative to 1 kG allele frequency, with absolute frequency differences greater than 5% indicated in bold text, and those with p values <0.1 indicated with an asterisk.

a For each polymorphism, the minor allele is listed first.

v SNPs verified by direct genotyping are indicated. The annotated list of all *ST8SIA2* variation identified is given in Table S1.

To examine SNPs that may affect post-transcriptional regulation, we examined SNPs that were within *ST8SIA2* untranslated regions, according to Refseq annotation. These SNPs may impact RNA-binding proteins, which are known to play a crucial role in RNA localisation, stability and splicing of neuronally expressed transcripts, which is in turn critical during brain development [46]. We identified two SNPs in the 5′UTR (rs3743365, rs3743364), and 10 SNPs within the 3′UTR, including one variant rs2290492 that lies ∼350 bp past the stop codon, near predicted miRNA binding sites [47]. A large proportion of the *ST8SIA2* 3′UTR proximal to the stop codon is conserved and we found two SNPs in the 3′UTR with GERP >2 in conserved elements (rs139149207 and rs2290492).

### Haplotype-specific functional variants

Finally, we examined how SNPs segregated with haplotypes in *ST8SIA2* that were associated with mental illness. We considered the 376 SNP loci genotyped by GATK or Sanger. Of these, 347 were successfully phased and thus could be assessed for whether variants segregated with the risk, the protective or other haplotypes.

To look for variants that could confer the risk or protective effect of our previously identified haplotypes in isolation, we looked for variants that resided exclusively on the risk or protective haplotypes and not other haplotypes. We found that 19 variants (seven novel) lay exclusively on the risk haplotype (MAF  = 0.011–0.024), and five variants (one novel) lay exclusively on the protective haplotype (MAF  = 0.011–0.025). Of those on the risk haplotype, two are transcribed (rs115781738 and rs145948851), and five were within DHSPs (rs117156502, rs112804054, rs115781738, 92937845 and 92973414). The latter two of these sites were conserved with GERP >2. The minor alleles of five SNPs were found exclusively on the protective haplotype: one of these was novel (at base position 92956314) and one localised within a DHSP (rs141457407). All of the SNPs that reside exclusively on the risk or protective haplotype are rare, and therefore cannot individually account for the increased risk or protective effect.

A recent study demonstrated that clusters of common disease-associated variants, located within multiple enhancer elements in *cis*, can act in combination to regulate gene expression and increase disease risk [32]. Alleles of such SNPs would contribute to a risk or protective effect when inherited together with other regulatory alleles in a specific haplotype, but would not necessarily have a discernible regulatory effect when inherited as part of other haplotypes. To attempt to capture this class of variant, we looked for alleles that segregated with either the risk or the protective haplotype in at least one chromosome, regardless of segregation with other haplotypes in additional chromosomes, and intersected these variants with *ST8SIA2*-specific PreSTIGE enhancer predictions [32], which existed for two cell lines: neuronal precursor cells (NPC) and human skeletal muscle myoblasts (HSMM), the locations of which are indicated in Figure 5. We expected that alleles having a discernible effect via this mechanism would be relatively common and present in the general population, so we used phased genotypes from 1 kG CEU and GBR population panels [28], which provide a larger population sample than our bipolar cohort.

Of 348 chromosomes in the 1 kG individuals, 127 carried the risk haplotype and 42 carried the protective haplotype. Seventy-eight SNPs segregated with the *ST8SIA2* 6-SNP risk haplotype in ≥ 1 individual and the protective haplotype in 0 individuals, including two SNPs which were used to tag the originally identified *ST8SIA2* risk haplotype. Eight SNPs were tagged by two of six SNPs constituting the risk haplotype (rs2035645 and rs4777974). Of these, three are in PreSTIGE enhancer elements (rs11074066, rs921846 and rs722645), one is also in a DHSP in hESC cells (rs921846) and one is in a foetal brain tissue DHSP cluster (rs722645). Conversely, 40 SNPs segregated with the protective haplotype in ≥ 1 individual and the risk haplotype in 0 individuals. Twenty-five SNPs were tagged by four of six SNPs constituting the protective haplotype (rs4586379, rs11637898, rs11074070 and rs3784735). Of the tagged SNPs, 13 were in PreSTIGE enhancers. Although none of these 13 were in DHSPs from hESC or neuronal cell lines, two (rs11074064 and rs11074065) were in a foetal brain tissue (DHSP) cluster.

### Validation and association of putative functional variants

We selected a subset of putative functional variants for validation and association analysis via direct genotyping, in a larger cohort of bipolar cases and control individuals (n = 215 cases, including the 47 sequenced individuals; and n = 165 controls). Variants were selected for Sequenom assay design from those that: 1) lie within PreSTIGE enhancer elements or DHSPs; 2) have a GERP score >2; 3) have an allele frequency difference between bipolar and 1 kG populations of >0.05. Of the 57 SNPs selected, 12 failed assay design or quality control stages, leaving 45 SNPs successfully genotyped via Sequenom (14 novel, 31 known). We also performed manual genotyping of three 3′UTR SNPs via restriction assay (rs2290492 and rs1869774) or direct sequencing (rs8035760).

The concordance rate for Sequenom-derived versus 454-derived genotypes was 97%, just below the concordance rate from Illumina genotyping assay validation. For manually genotyped variants, we found that rs8035760 had a low rate of concordant genotype calls (56%) compared to the other 3′UTR variants (97%). This was likely due to erroneous rs8035760 genotype calls from 454 sequencing, consistent with a low SNP call quality (QD = 6) and proximity of the site to a short homopolymer in the human reference sequence (data not shown).

Eleven of the 14 novel variants (79%) were validated in one or more samples (Table 5). Of the three SNPs that were not validated, two were called by Refmapper only whereas the third was called by both GATK and Refmapper. We also found additional cases from the larger cohort who were heterozygous for one of four of the genotyped novel SNPs, including SNP 15∶92961050, which was found in an additional three cases (two bipolar type I and one bipolar type II), and no control individuals (uncorrected p = 0.079). This SNP is within a PreSTIGE putative enhancer region in intron 1. All remaining SNPs that were found in additional cases were also found in at least one unaffected control individual.

**Table 5:** Fourteen novel SNPs assayed by Sequenom for verification.

Base Position (hg19)	Reference Allele	Alternative Allele	AC in Affected (n = 215)	AC in Unaffected (n = 165)	Set
11: 113102360	T	NP	0	0	3a
15: 92926056	G	A	2	0	1
15: 92929229	G	C	1	0	1
15: 92956314	A	G	1	0	1
15: 92958227	C	G	1	0	1
15: 92958793	C	A	1	0	1
15: 92959424	A	NP	0	0	1
15: 92961050	A	G	4	0	3a
15: 92961385	A	G	1	1	1
15: 92973414	G	A	1	0	1
15: 92992055	C	T	2	0	1
15: 92993375	C	T	2	1	1
15: 92994038	T	NP	0	0	3a
15: 92994449	G	A	1	1	1

Three SNPs that were detected in 454-generated sequence were non-polymorphic (NP) after direct genotyping. The allele count (AC) is given from a total of 380 subjects (215 cases including the 48 sequenced individuals, 165 controls). The algorithm set from which the SNP was observed is provided (1 =  GATK & Refmapper; 2 =  GATK only; 3a  =  Refmapper only; 3b  =  Refmapper only & filtered in GATK; 4 =  Sanger).

Next, we performed a test of association with bipolar disorder (n = 215 cases, 165 controls). Thirteen subjects were excluded from association analysis due to high rates of genotype missingness (>10%; five cases and five controls). Two SNPs were nominally associated (rs11074064 & rs722645, p<0.05; Table 6) and lie in an LD block within intron 1 (chr15∶92,940,731–92,944,864; Figure S2), although these SNPs did not remain significant after multiple testing correction for 11 independent tests. Interestingly, both rs11074064 and rs722645 were among those found to segregate only with the protective or risk haplotype, with the same direction of effect as the protective and risk alleles respectively. Both rs11074064 and rs722645 also lie within the same PreSTIGE enhancer element spanning the TSS and part of intron 1 and are present within each of two neighbouring DHSPs in foetal brain (data not shown). Thus, these variants are plausible candidates for functional SNPs that contribute to the effect of the protective or risk haplotypes respectively.

**Table 6:** SNPs nominally associated with bipolar disorder.

SNP	base Position (hg19)	Alt allele	Freq (Affected)	Freq (Unaffected)	Ref allele	P-value	Odds ratio (95%CI)
rs11074064	92944662	A	0.423	0.509	G	0.019	0.71 (0.53–0.95)
rs722645	92943323	G	0.459	0.381	A	0.035	1.38 (1.02–1.85)

The single nucleotide polymorphisms (SNP) nominally associated with bipolar disorder (p<0.05) are given. The SNP position on chromosome 15 is given relative to hg19 build. The reference (Ref) and alternative (Alt) alleles are shown, with the frequency (Freq) of the alternative allele in affected individuals (n = 210 cases) and unaffected controls (n = 160) shown. Odds ratio of effect and the 95% confidence interval (CI) are given.

## Discussion

There is growing evidence of the involvement of *ST8SIA2* in risk of mental illness, with the association of variants within the gene with bipolar disorder [20], schizophrenia [21], [22] and autism [25] (reviewed in [48]). Post-mortem studies also implicate altered levels of PSA-NCAM in brains of people with bipolar disorder, schizophrenia and major depression [9]–[11]. Formation of PSA-NCAM is critical for fibre tract formation, neurogenesis and plasticity (reviewed in [4]), and is dependent on availability and functionality of both enzyme and substrate. Animal studies that have begun to assess combinations of mutant alleles, have shown the amount of NCAM devoid of PSA during brain development is critical, and highlights the important balance between these molecules for brain development and function [7], [49] (reviewed in [50]). Further, loss-of-function mutations have previously been reported in *ST8SIA2* in schizophrenia [21], [51]. Hence, we conducted a targeted resequencing study to identify genetic variants which may impact formation of PSA-NCAM in bipolar disorder. We targeted both *ST8SIA2* and the region of interaction in its principal substrate NCAM1 [33]–[35]. *NCAM1* has also shown genetic association with bipolar disorder [23], schizophrenia [52] and neurocognition [53].

In any sequencing study, it is important that the coverage is adequate to be confident that all detectable nucleotide variation is identified. We performed a number of tests for quality control in our data to ensure that the nucleotide diversity we report herein is accurate and complete. We used GATK, which has so far been predominantly used for genome or exome-wide studies, as our principal method for diploid genotype calling. Using the probabilistic genotyping module within GATK resulted in high genotype accuracy, even when including genotypes called on sites with low coverage. Diploid genotyping is critically important for detailed genetic analysis, including haplotype reconstruction and accurate assessment of allele frequency within disease or general populations, and relies on genotypes that are as complete and accurate as possible given the current limitations of sequencing data. In comparison, the use of a simple threshold approach to distinguish between homozygote and heterozygote variants requires a high level of coverage for accurate heterozygote calling. With this approach many regions that have systematically low coverage with current sequencing technology would be missed, which would particularly affect the identification and genotyping of rare variants. Despite our best efforts to equalise coverage across every base pair in every individual, we acknowledge that important loss-of-function variants may exist in *ST8SIA2* which were not identified in the current study, due to low coverage of some genomic regions in particular individuals.

We identified 72 variants in our study that were not observed in the 1 kG Caucasian population (41 novel and 26 known SNPs in *ST8SIA2*, 5 novel SNPs in *NCAM1*). Unfortunately, we were not able to compare the rates of novel variant detection between the bipolar and 1 kG populations due to inherent differences in coverage and platform – which is a major limitation of the study. However, as the majority of novel SNPs were observed in a small number of cases, we can deduce that they cannot individually account for the increased risk attached to the specific risk haplotype previously identified [20]. Indeed, the majority of variants may have arisen on a spectrum of haplotypes – not restricted specifically to the risk haplotype previously associated with illness – consistent with expectations of allelic heterogeneity, which confounds identification of disease-causing variants.

We observed no mutations in the protein-coding regions of either *ST8SIA2* or *NCAM1* that would reduce functionality of the enzyme or its substrate protein. Therefore, we focused our analysis on prediction of function in non-protein-coding gene regions. The enrichment of SNPs at conserved positions in the genome within DHSP sites in our data is consistent with rare and deleterious variants being present in *ST8SIA2*. Although measures of cross-species conservation are useful in highlighting variant sites that are most likely to be functional, not all functional residues are expected to have been conserved across species evolution. Despite the wealth of data available on DHSs, most conserved sites have no obvious predictable functional effect. This may be indicative of the limitations of the currently available cellular models, or current knowledge of modes of gene regulation. In the absence of any clear predicted function of these conserved sequences, forward genetics techniques could be used to examine the impact of these variants.

In addition to rare and highly deleterious variants, common variants that have a more subtle effect on *ST8SIA2* function are also plausible candidates for contributing to increased risk of mental illness by regulating the timing and level of gene expression. The dose-responsive and functionally redundant nature of *ST8SIA2* and *ST8SIA4* is well characterised [7], and it is possible that genetic interaction between these two genes determines risk, with the dosage of each gene contributing. Sites within the 3′UTR are particularly attractive candidates for such an effect, given the important role of microRNAs in fine-tuning gene expression in the brain [54]. MicroRNA binding sites are involved in exquisite control of gene dosage during differentiation, a process that if disrupted may lead to a subtle neurodevelopmental phenotype that may precede bipolar disorder symptomatology. We found two SNPs within a conserved region at the start of the 3′UTR that are strong candidates for altering the post-transcriptional regulation of *ST8SIA2*. Common variants that impart an effect on sites of transcriptional regulation are also candidates for imparting subtle gene-regulatory effects. A recent finding implicated a combinatorial effect of multiple enhancers on gene expression and, in turn, genetic risk of disease [32]. Intriguingly, in that study, *ST8SIA2* was identified as a bipolar GWAS locus in which variants in enhancers functionally impact expression in neuronal precursor cells [32]. Although the GWAS result [24] was not obtained in the same population as our cohort, this finding suggests that the combination of multiple DNA variants across multiple enhancers is an important mode of regulation at the *ST8SIA2* locus in bipolar disorder. This pattern of risk inheritance is entirely consistent with a combinatorial effect of many risk variants that lie on alternative haplotypic backgrounds, as we indicate.

In the current study, we identified two variants with putative functional effects that are nominally associated with disease and were found to segregate with the either the protective or the risk haplotype (rs11074064 and rs722645). Under the model of a combinatorial effect from multiple enhancers, these variants, in combination with other functional variants inherited with a specific haplotype, could confer the functional effect of the risk and protective haplotypes. As these variants would be expected to alter gene expression, further work combining close genetic characterisation at the *ST8SIA2* locus with gene expression data could be used to effectively pinpoint the genetic determinants of this potentially critical mode of gene regulation.

While we have not identified obvious loss-of-function mutations that would affect the formation of PSA-NCAM in this cohort of patients with bipolar disorder, we have characterised the nucleotide variation that may be associated with the disease, and importantly, in the context of the specific risk and protective haplotypes previously identified. Further analysis of the functional impact of these candidate variants on the regulation of gene expression *in vitro* will likely elucidate further insights into the mechanisms through which this candidate gene increases risk to mental illness.

## Materials and Methods

### Ethics statement

This study was carried out in accordance with the latest version of the Declaration of Helsinki after specific approval of the study and consent procedures by the University of New South Wales Human Research Ethics Committee (HREC # 04144 and 10078). All participants provided informed written consent prior to inclusion in the study.

### Cohort selection

Individuals were selected from the Australian bipolar disorder case-control cohort, which has been described previously [20]. Diagnosis was made by structured interview using the Diagnostic Interview for Genetic Studies (DIGS), using Research Diagnostic Criteria. To enrich for individuals who may have a genetic defect in *ST8SIA2*, we selected one individual affected with bipolar disorder type 1 (BPI) or type 2 (BPII) (n = 39 and n = 3 respectively) from 42 families who showed evidence of linkage to chromosome 15q25–26 [16], and six additional unrelated BPI cases from our case-control cohort to ensure equivalent representation of major haplotypes to those observed in population samples [20]. A total of 45 BPI cases (94%) and three BPII cases (6%) formed the selected sample.

### 454 PCR enrichment and Sequencing

We used long-range PCR to amplify genomic DNA from the target regions prior to 454 sequencing, dividing the *ST8SIA2* locus into eight amplicons and covering the *NCAM1* region of interest in a single amplicon.

Although we initially attempted to enrich the entire *ST8SIA2* locus in this manner, we could not amplify a region surrounding the *ST8SIA2* transcription start site, likely owing to a high GC content at the intended primer binding site (Figure 1). This GC-rich region was also found to be less accessible to next generation sequencing in an analysis of accessible regions reported in the 1 kG project (Figure 1). After several optimisation attempts (primer sequences: F-AAAGTTACAAAAGGAATGCAAATG, R-AGGAACCTCGACTGCCAAC; F-CCCACTACCACTTCCAGCTC, R-GCAGAGCAGTTTGGGAAGTC and F-CCCCCTTCAGAGATGATGAA, R-GCAGAGCAGTTTGGGAAGTC) this gap region was divided into two subregions. The 3′ subregion was successfully amplified by long-range PCR (ST8-2), but the 5′ subregion could not be amplified (primer sequences F-CCCCCTTCAGAGATGATGAA R-AAAGAAGCAGGGAGCACTCA). In order to effectively examine nucleotide variation within this region, we divided the unamplified region into 8 overlapping short-range PCR amplicons for subsequent bi-directional Sanger sequencing (Figure 1).

Final primer sequences (5′ to 3′) for long-range PCR were as follows: ST8-1F-AGCGTCTTTTCAGGAGGTGA; ST8-1R-CTACCCTGACCCAGCAACAT; ST8-2F-CGCTTCCTGCTCTCATTTTC; ST8-2R-GTTTCCTCCTTGCCATCGT; ST8-3F-TCCCAGTGAAGAGCACAGTC; ST8-3R-TTCCCATTGCCCTGAGTATC; ST8-4F-TAAGGTGGAGCTAGGGACCA; ST8-4R- GGAGACCAAATGCCTTGAAA; ST8-5F-TCCCATCTGTGATTCCATGA; ST8-5R-GGGCAGGATCTTCTTTCTCC; ST8-6F-AATGACAAGTGCCCCATAGC; ST8-6R-ACTGCAAACTCATGCTGACAA; ST8-7F-ACCCACTTTTTCTCCCCAGT; ST8-7R-TATGAGCACGGACAGCAATC; ST8-8F-ATCAGCCTTTCCCAACAGC; ST8-8R-CCACTCCCTCTACCCAATTTC; N1F-CATCTTTCAACCAACCAGCA; and N1R-CCTATTCCCCTCCAGCTACC.

Long-range PCR was performed using the Expand Long Range dNTPack kit (Roche, Mannheim, Germany), according to manufacturer's instructions. Briefly, 50 μl reaction volumes consisted of 10 μl of 5x Expand Long Range Buffer with 12.5 mM MgCl_2_, 2.5 μl of 10 mM PCR Nucleotide Mix, 0.3 μM of each primer, 0.7 μl of 5 U/μl Expand Long Range Enzyme Mix, with 500 ng genomic DNA. DMSO (5%) was added to PCR mix of all amplicons except amplicons ST8-2 and ST8-3. Thermal cycling conditions were performed on a PTC-240 DNA Engine Tetrad 2 Cycler (MJ Research, Bio-Rad Laboratories, Copenhagen, Denmark) provided by the manufacturer. The annealing temperature was 60°C for all amplicons, except amplicons ST8-3 and ST8-5 which were 59°C and 58°C, respectively.

PCR products from each amplicon were quantified by comparison to DNA molecular weight marker XV (Roche, Mannheim, Germany) after gel electrophoresis, using Bio-Rad Quality One software and Molecular Imager ChemiDoc XRS System (Bio-Rad, Hercules, CA, USA). Individual PCR products from the nine amplicons for each individual were purified using MultiScreen_96_-PCR filter plate (Millipore, Bedford, MA), and resuspended in 30 μl Milli-Q-grade water. Quality and concentration was checked by Nanodrop 1000 Spectrophotometer (Biolab, Musgrave, VIC, Australia). After concentration normalisation, all amplicons for each individual were pooled at an equimolar ratio (approx. 5 ng/μl each) to generate 1 μg of template for library preparation, which was performed by the Ramaciotti Centre for Gene Function Analysis (University of New South Wales, Sydney, Australia). Sequencing was performed on the 454 GS-FLX (454 Life Sciences, Branford, CT, USA).

### Sanger Sequencing

Overlapping primers were designed across the target region (hg19: chr15∶92933439-92938793 bp), based on overlapping amplicons of at least 500 bp, using the PCR suite [55] and Primer3 [56]. Primers used were 1L-ACGTGCGGAAGAGGATATTG; 1R-ATTTTCCAAGAAGCCCGAAG; 2L-GGCCTTTAATTTGCCACCAC; 2R-AGGTGACCCACGATTACAGC; 3L-TGGGTGCAAAGAAGGAAGAC; 3R-TGCTTGAAGGGTGGTTTAGG; 4L-GCGCATCCTGTACTTTCCTC; 4R-TTATCCCCTGGACCACTCAG; 5L-GGGCACAAGCAGTACTTTGG; 5R-GGCAGATGTTTCAGCAGTTG; 6L-CGCCCACAAACTGTAGTCAG; 6R-CGGAGGGTGGAGAGTACAGA; 7L-GAAATCGGGTAAATAGCTGCTC; 7R-GCGAGGTCGGGAGAAA; 8F-GGAGACGGAGACATTTCGGG; 8R-GCTCTTGGGGAAACCGAGAA. Genomic DNA was amplified using Platinum Taq (Invitrogen, Carlsbad, CA) and purified using Multiscreen_96_-PCR plate. Sequencing reactions were performed in both forward and reverse directions for each amplicon, using the Big Dye Terminator v3.1 Cycle Sequencing Kit (Applied Biosystems, Foster City, CA, USA). The extension products were analysed on an ABI 3730 by the Ramaciotti Centre (University of New South Wales, Australia). The Phred/Phrap/Polyphred software suite [41] was used for base calling, sequence alignment and polymorphism identification. Consed [42] was used for viewing sequences and traces, and to confirm polymorphisms in each individual by their presence on both forward and reverse sequences.

### Data Acquisition and Processing

dbSNP 135 vcf files were downloaded from the NCBI ftp server (ftp://ftp.ncbi.nih.gov/snp/organisms/human_9606/VCF/), and the subset of variants within the sequenced region was selected using SelectVariants and CombineVariants modules of the GATK. The 1 kG reference panel in BEAGLE format was downloaded from http://bochet.gcc.biostat.washington.edu/beagle/1000_Genomes.phase1_release_v3/. A subset of reference panel genotypes, including Caucasian European individuals (CEU and GBR populations) and sequence target region markers, was selected from the reference panel using BEAGLE utilities (http://faculty.washington.edu/browning/beagle_utilities/utilities.html). Genotype data for 1 kG Caucasian European Individuals was downloaded using the 1000 Genomes Data Browser.

### 454 Analysis: Mapping

BWA mapping: SFF files containing chromatograms were converted to fastq files using sff2fastq (https://github.com/indraniel/sff2fastq). Fastq sequences were demultiplexed using appropriate RLMID barcodes for each sample and the barcode sequences were subsequently trimmed using the FASTX toolkit (http://hannonlab.cshl.edu/fastx_toolkit/). Reads were aligned to the b37 Human Genome Reference Consortium human reference sequence, downloaded from Broad Institute (http://www.broadinstitute.org/gatk/guide/article.php?id=1213). Alignment was performed using BWA [36] in Smith-Waterman mode resulting in sam files [57]. Readgroup meta information, necessary for combined analysis of individual samples in GATK [40], was added and files were merged into a single bam file using picardtools (http://picard.sourceforge.net/).

Refmapper: Reads were aligned to fasta files of chromosomes 11 and 15 of the hg19 UCSC human reference sequence using Refmapper (version 2.6, 454 Life Sciences, Branford, CT, USA).

### 454 Analysis: SNP calling and Genotyping

GATK: The GATK modules CountCovariates and TableRecalibration were used for basecalling recalibration. When applying default covariates, we found that base call quality scores did not vary predictably when stratified across machine cycle, which is in contrast to findings from whole-genome data [39], and is likely due to the smaller size of our targeted sequencing dataset. To avoid potentially introducing artefact, we excluded this covariate during base call quality recalibration within our analysis.

Following recalibration, UnifiedGenotyper was used for SNP calling and genotyping. SNPs were then filtered using VariantFiltration module based on Best Practice Variant Detection V4 recommended hard filters (http://www.broadinstitute.org/gatk/guide/article?id=1186), these were QD (Quality By Depth) <2.0 (GATK recommended) or 5.0 (our analysis), MQ (Mapping Quality) <40.0, FS (Fisher Strand Bias) >60.0, HaplotypeScore (Haplotype Score) >13.0, MQRankSum (Mapping Quality Rank-Sum p value) <−12.5, ReadPosRankSum (Read Position Rank Sum Test) <−8.0. A custom Galaxy workflow incorporating these steps, which are based on the Best Practice Variant Detection v3 is available (https://main.g2.bx.psu.edu/u/a-shaw-neura/p/next-generation-resources).

Refmapper: SNP calling was performed by merging the HCDiffs.txt output from Refmapper for the 48 individually mapped individuals.

### Genotyping and Allele Frequency Comparison

Illumina genotype data was prepared using PLINK-Seq (http://atgu.mgh.harvard.edu/plinkseq/) from two association studies of Australian bipolar disorder case-control cohort samples: one using an Illumina BeadArray [20] and one using an Illumina 660 W chip [43]. Overlapping sample genotypes from the two datasets were checked for exact concordance using GATK VariantEval module before merging into a single set of comparison genotypes. Concordance between Illumina genotypes and sequencing genotypes was examined using GATK VariantEval module.

1 kG Caucasian European comparison: SNPs identified in both datasets or unique to each dataset were selected using the SelectVariants GATK module. The sets of unique or overlapping SNPs were then compared using GATK VariantEval module. SNPs that were co-located with indels identified in 1 kG, and SNPs identified in Refmapper but not GATK, were not included in the AF analysis, as the AF at these sites could not be compared across the two datasets. A custom Galaxy workflow incorporating the above steps is available at https://main.g2.bx.psu.edu/u/a-shaw-neura/p/next-generation-resources.

### BEAGLE Imputation and Phasing

Haplotypes were phased and genotype calls and quality scores were adjusted using GATK and BEAGLE, as per the example usage (http://gatkforums.broadinstitute.org/discussion/43/interface-with-beagle-software). Genotype likelihoods from 454 were prepared for BEAGLE input using ProduceBeagleInput GATK module. These likelihoods were then merged with genotype data from Sanger-sequenced region and Illumina genotypes, with Illumina genotypes taking priority over sequencing calls for discordant genotypes. BEAGLE was run using default parameters, with or without additional genotypes from a 1 kG reference panel as input. Phased genotypes were then converted into GATK vcf format using the BeagleOutputToVCF GATK module.

### Bioinformatic analysis of predicted function

DNAse hypersensitivity site uniform peaks (DHSPs) from the ENCODE project [31], [45] were downloaded from UCSC Genome Browser [58]. Peaks from hESC (h1 and h7 lines) and neuronal (SK-N-SH RA+, SK-N-MC and BE(2)C lines) were each downloaded into the Galaxy suite. For foetal brain, DHSPs from nine library preparations from six donors were downloaded from the Roadmap Epigenomics Project [59] browser. The peaks were clustered in Galaxy to eliminate peaks that were only present in a single library preparation. Genome Evolutionary Rate Profiling (GERP) scores were downloaded from the UCSC Genome Browser for each variant site, and GERP conserved elements were obtained from http://mendel.stanford.edu/SidowLab/downloads/gerp/. Splice site predictions were performed using two web-based tools: NNSPLICE (http://www.fruitfly.org/about/index.html) [60], and Human Splicing Finder V2.4.1 (http://www.umd.be/HSF/) [61]. Variant Effect Predictor (http://asia.ensembl.org/info/docs/variation/vep/index.html) [62] was used to identify putative functional variants within the coding regions. PreSTIGE Enhancer regions for *ST8SIA2* were downloaded from http://genetics.case.edu/prestige/.

### Sequenom Validation

SNPs were selected for Sequenom validation based on the following criteria: Criteria 1 ([GERP >2] and [in DHS or PreSTIGE Enhancer or Frequency difference ≥ 0.05 between 454 bipolar and 1 K genomes CEU]); and Criteria 2 ([in PreSTIGE Enhancer or DHS] and [Frequency difference ≥ 0.05 or novel]). Assays for selected SNPs were designed and performed at the Australian Genome Research Facility (University of Queensland, Brisbane, Australia).

### Manual Genotyping and Association Analysis

For three SNPs, manual genotyping by RFLP or Sanger sequencing was performed in Australian bipolar disorder case-control cohort individuals. Merging of manual genotypes and Sequenom genotypes and subsequent statistical analyses were performed using PLINK software. SNPs with MAF <0.05 were removed prior to association analysis. Association analysis for bipolar disorder was performed using a broad disease model where individuals diagnosed with BPI, SZMA, BPII or MDD were all classified as affected (n = 215).

### Web resources

1000 Genomes Data Browser (http://browser.1000genomes.org/index.html)

1000 genomes reference panel in BEAGLE format (http://bochet.gcc.biostat.washington.edu/beagle/1000_Genomes.phase1_release_v3/)

dbSNP 135 vcf files (ftp://ftp.ncbi.nih.gov/snp/organisms/human_9606/VCF/)

BEAGLE utilities (http://faculty.washington.edu/browning/beagle_utilities/utilities.html)

sff2fastq (https://github.com/indraniel/sff2fastq)

FASTX toolkit (http://hannonlab.cshl.edu/fastx_toolkit/)

Human Genome Reference Consortium b37 human reference sequence (http://www.broadinstitute.org/gatk/guide/article.php?id=1213)

picardtools (http://picard.sourceforge.net/)

PLINK-Seq (http://atgu.mgh.harvard.edu/plinkseq/)

NNSPLICE (http://www.fruitfly.org/about/index.html)

Human Splicing Finder V2.4.1 (http://www.umd.be/HSF/)

Variant Effect Predictor (http://asia.ensembl.org/info/docs/variation/vep/index.html).

PreSTIGE Enhancers (http://genetics.case.edu/prestige/)

GERP++ elements (http://mendel.stanford.edu/SidowLab/downloads/gerp/)

Roadmap Epigenomics Consortium browser (http://www.epigenomebrowser.org/)

UCSC Genome Browser (http://genome.ucsc.edu/)

TargetScan (http://www.targetscan.org)

A Galaxy workflow/pipeline for sequence analysis used in this study is available at: https://main.g2.bx.psu.edu/u/a-shaw-neura/p/next-generation-resources.

## Supporting Information

Figure S1 **Comparison of genotype quality score and minor allele frequency to identify potentially erroneous SNPs.** All points represent raw SNPs that were novel (not present in dbSNP 135). Allele frequency (AF) vs. Quality by Depth score (QD). Hollow diamonds – SNPs that failed filtering according to GATK recommended filtering, Black circles – SNPs that passed filtering. Points in dashed oval have low QD and high AF, suggesting they are not bona-fide SNPs.
(DOCX)

Figure S2 **Linkage disequilibrium plot of SNPs verified by direct genotyping in larger case control cohort (n = 207 bipolar cases, 160 controls).** Six of the seven SNPs in block 2 had a p>0.1. Haplotype analysis of the block 2 SNPs showed a trend for association (omnibus p = 0.077), with the major haplotype (red) more frequent in affected cases (F_A) than unaffected controls (F_U), representing a risk haplotype. The second most common haplotype (green) was more frequent in unaffected controls (F_U) than affected cases (F_A), representing a protective haplotype.
(DOCX)

Table S1 **Complete list of genetic variation identified in** ***ST8SIA2*** **.** The location of all identified variants on chromosome 15 ST8SIA2 region (hg19 build), with transcribed SNPs indicated with asterisks. SNPs identified exclusively on the risk haplotype are shown (0 =  not exclusive, 1 =  present on risk and other haplotypes, 2 =  present on risk haplotype only, ND  =  not determined). SNPs identified exclusively on the protective haplotype are shown (0 =  not exclusive, 1 =  present on protective and other haplotypes, 2 =  present on protective haplotype only, ND = not determined). The set from which the SNP was observed is given (1 =  GATK & Refmapper; 2 =  GATK only; 3a  =  Refmapper only; 3b  =  Refmapper only & filtered in GATK; 4 =  Sanger). Co-localisation with DNase I hypersensitivity site peaks (DHSPs; neuronal = 1; hESC = 2; foetal brain = 3) are given. Genomic Evolutionary Rate Profiling (GERP) scores are provided for each variant that is within a GERP-conserved element. The nature of the polymorphism in each cohort is given (with minor allele listed first). The minor allele frequency (mAF) of each variant in the 47 bipolar cases and 174 Caucasian individuals (CEU and GBR) from the 1000 Genomes Project (1 kG) are shown separately. The frequency difference (freqDIFF) was calculated relative to 1 kG allele frequency, and those with p values <0.1 indicated with an asterisk. aFor each polymorphism, the minor allele is listed first. v SNPs verified by direct genotyping are indicated.
(DOCX)

Note S1 **GATK SNP Filtering Threshold Note S1: GATK SNP Filtering Threshold.**
(DOCX)

Note S2 **Removal of data in regions of PCR allelic-bias.**
(DOCX)
