# Supplementary material for: Characterisation of Genetic Variation in ST8SIA2 and Its Interaction Region in NCAM1 in Patients with Bipolar Disorder
Source: PLoS One. 2014 Mar 20;9(3):e92556. doi: 10.1371/journal.pone.0092556 (PMC3961385; doi:10.1371/journal.pone.0092556)
Supplement: Figure S1 — Comparison of genotype quality score and minor allele frequency to identify potentially erroneous SNPs. All points represent raw SNPs that were novel (not present in dbSNP 135). Allele frequency (AF) vs. Quality by Depth score (QD). Hollow diamonds – SNPs that failed filtering according to GATK recommended filtering, Black circles – SNPs that passed filtering. Points in dashed oval have low QD and high AF, suggesting they are not bona-fide SNPs. (DOCX) [file pone.0092556.s001.docx]

“Characterisation of genetic variation in *ST8SIA2* and its interaction region in NCAM1 in patients with bipolar disorder”

**AD Shaw, Y Tiwari, W Kaplan, A Heath, PB Mitchell, PR Schofield, JM Fullerton**


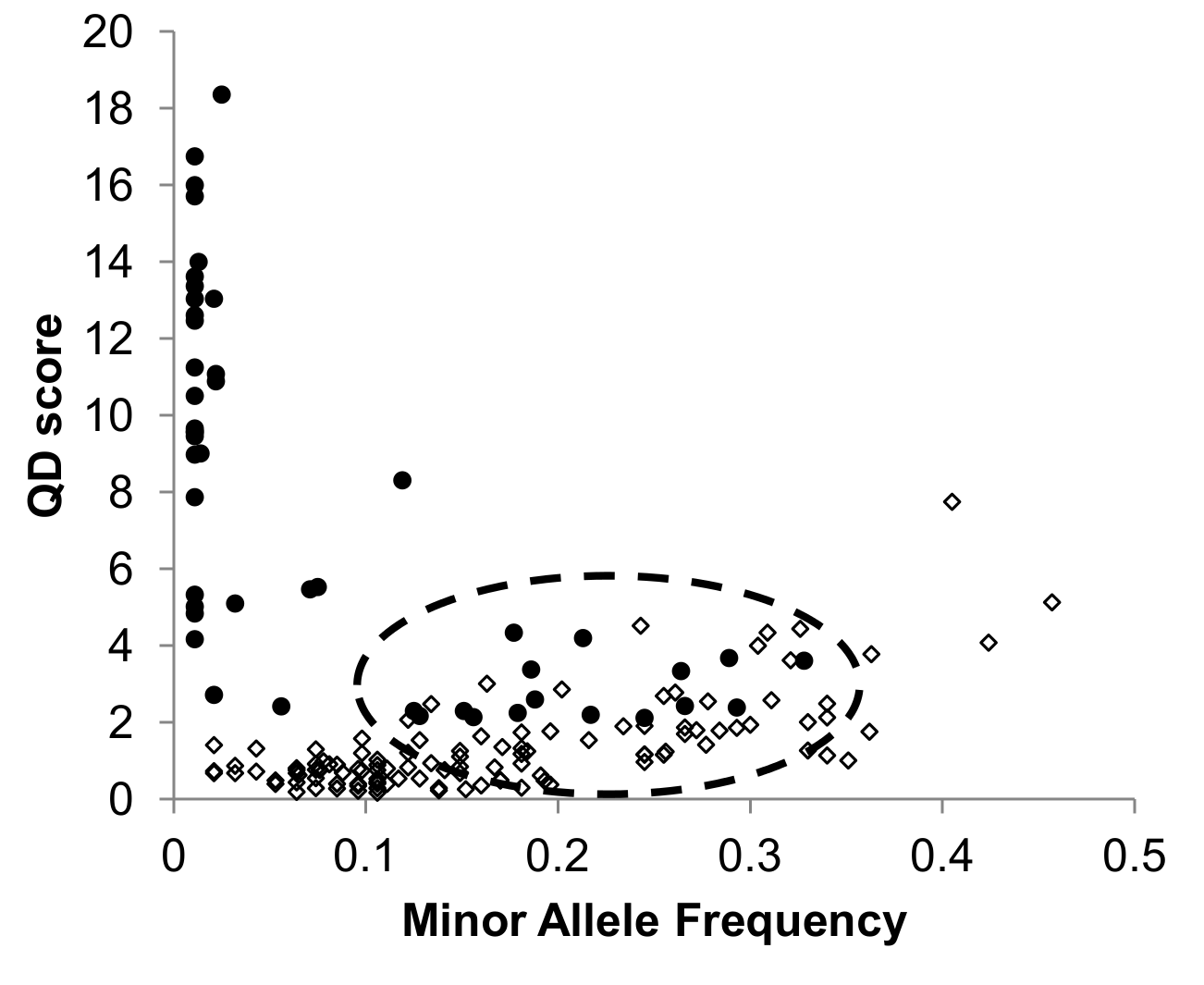


**Figure S1: Comparison of genotype quality score and minor allele frequency to identify potentially erroneous SNPs.** All points represent raw SNPs that were novel (not present in dbSNP 135). Allele frequency (AF) vs. Quality by Depth score (QD). Hollow diamonds – SNPs that failed filtering according to GATK recommended filtering, Black circles – SNPs that passed filtering. Points in dashed oval have low QD and high AF, suggesting they are not bona-fide SNPs.
