# Supplementary material for: Characterisation of Genetic Variation in ST8SIA2 and Its Interaction Region in NCAM1 in Patients with Bipolar Disorder
Source: PLoS One. 2014 Mar 20;9(3):e92556. doi: 10.1371/journal.pone.0092556 (PMC3961385; doi:10.1371/journal.pone.0092556)
Supplement: Figure S2 — Linkage disequilibrium plot of SNPs verified by direct genotyping in larger case control cohort (n = 207 bipolar cases, 160 controls). Six of the seven SNPs in block 2 had a p>0.1. Haplotype analysis of the block 2 SNPs showed a trend for association (omnibus p = 0.077), with the major haplotype (red) more frequent in affected cases (F_A) than unaffected controls (F_U), representing a risk haplotype. The second most common haplotype (green) was more frequent in unaffected controls (F_U) than affected cases (F_A), representing a protective haplotype. (DOCX) [file pone.0092556.s002.docx]

“Characterisation of genetic variation in *ST8SIA2* and its interaction region in NCAM1 in patients with bipolar disorder”

**AD Shaw, Y Tiwari, W Kaplan, A Heath, PB Mitchell, PR Schofield, JM Fullerton**

**
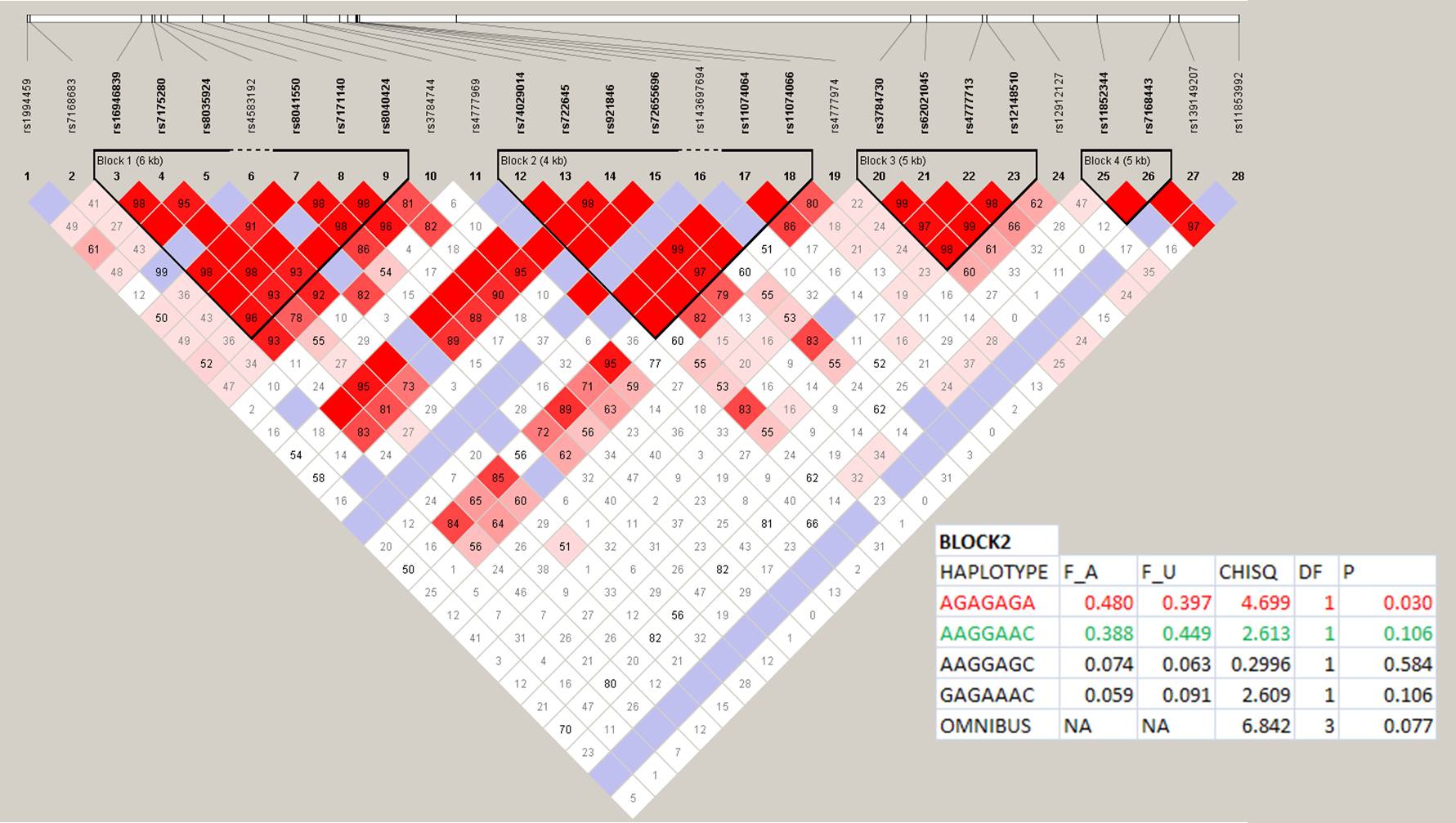
**

**Figure S2: Linkage disequilibrium plot of SNPs verified by direct genotyping in larger case control cohort (n=207 bipolar cases, 160 controls).** Six of the seven SNPs in block 2 had a p > 0.1. Haplotype analysis of the block 2 SNPs showed a trend for association (omnibus p=0.077), with the major haplotype (red) more frequent in affected cases (F_A) than unaffected controls (F_U), representing a risk haplotype. The second most common haplotype (green) was more frequent in unaffected controls (F_U) than affected cases (F_A), representing a protective haplotype.
