# Supplementary material for: Characterisation of Genetic Variation in ST8SIA2 and Its Interaction Region in NCAM1 in Patients with Bipolar Disorder
Source: PLoS One. 2014 Mar 20;9(3):e92556. doi: 10.1371/journal.pone.0092556 (PMC3961385; doi:10.1371/journal.pone.0092556)
Supplement: Table S1 — Complete list of genetic variation identified in ST8SIA2 . The location of all identified variants on chromosome 15 ST8SIA2 region (hg19 build), with transcribed SNPs indicated with asterisks. SNPs identified exclusively on the risk haplotype are shown (0 = not exclusive, 1 = present on risk and other haplotypes, 2 = present on risk haplotype only, ND = not determined). SNPs identified exclusively on the protective haplotype are shown (0 = not exclusive, 1 = present on protective and other haplotypes, 2 = present on protective haplotype only, ND = not determined). The set from which the SNP was observed is given (1 = GATK & Refmapper; 2 = GATK only; 3a = Refmapper only; 3b = Refmapper only & filtered in GATK; 4 = Sanger). Co-localisation with DNase I hypersensitivity site peaks (DHSPs; neuronal = 1; hESC = 2; foetal brain = 3) are given. Genomic Evolutionary Rate Profiling (GERP) scores are provided for each variant that is within a GERP-conserved element. The nature of the polymorphism in each cohort is given (with minor allele listed first). The minor allele frequency (mAF) of each variant in the 47 bipolar cases and 174 Caucasian individuals (CEU and GBR) from the 1000 Genomes Project (1 kG) are shown separately. The frequency difference (freqDIFF) was calculated relative to 1 kG allele frequency, and those with p values <0.1 indicated with an asterisk. aFor each polymorphism, the minor allele is listed first. v SNPs verified by direct genotyping are indicated. (DOCX) [file pone.0092556.s003.docx]

“Characterisation of genetic variation in *ST8SIA2* and its interaction region in NCAM1 in patients with bipolar disorder”

**AD Shaw, Y Tiwari, W Kaplan, A Heath, PB Mitchell, PR Schofield, JM Fullerton**

**Table S1: Complete list of genetic variation identified in *ST8SIA2*.**

| SNP | Chr 15 position | novel | mA Excl. to RISK | mA Excl. to PROT | Set | DHSP | GERP | poly^a^ | Freq (BP) | Freq (1kG) | freqDIFF |
| --- | --- | --- | --- | --- | --- | --- | --- | --- | --- | --- | --- |
| rs1994459 ^v^ | 92919290 |  | ND | ND | 3b |  | 0.59 |  | ND | 0 | **ND** |
| rs7168683 ^v^ | 92919518 |  | 0 | 0 | 1 |  |  | G/A | 0.12 | 0.126 | -0.007 |
| rs4777708 | 92919771 |  | 1 | 0 | 1 |  |  | C/A | 0.12 | 0.126 | -0.007 |
| rs1386772 | 92919833 |  | 1 | 0 | 1 |  |  | T/C | 0.13 | 0.118 | 0.013 |
| rs7174226 | 92919891 |  | 1 | 0 | 1 |  |  | C/T | 0.12 | 0.129 | -0.01 |
| rs2387805 | 92919986 |  | 1 | 0 | 1 |  |  | G/T | 0.163 | 0.17 | -0.007 |
| rs1386773 | 92920023 |  | 0 | 1 | 1 |  |  | T/C | 0.217 | 0.207 | 0.011 |
| **rs1386774** | **92920058** |  | **1** | **0** | **1** |  |  | **T/C** | **0.568** | **0.218** | **0.349#** |
| rs112724220 | 92920135 |  | 0 | 0 | 1 |  |  | C/G | 0.011 | 0.009 | 0.003 |
| rs4299104 | 92920197 |  | 1 | 0 | 1 |  |  | C/G | 0.107 | 0.126 | -0.019 |
| 15:92920362 | 92920362 | Y | ND | ND | 3b |  |  |  | ND | 0 | ND |
| rs1075296 | 92920364 |  | 1 | 0 | 1 |  |  | G/A | 0.109 | 0.126 | -0.018 |
| rs78124821 | 92920408 |  | 1 | 0 | 1 |  |  | T/G | 0.022 | 0.043 | -0.021 |
| rs12908291 | 92920826 |  | 1 | 0 | 1 |  |  | A/G | 0.065 | 0.066 | -0.001 |
| rs2101436 | 92920921 |  | 1 | 0 | 1 |  |  | T/C | 0.12 | 0.126 | -0.007 |
| rs7175122 | 92920949 |  | 1 | 0 | 1 |  |  | T/C | 0.043 | 0.032 | 0.012 |
| rs4586379 | 92921027 |  | 0 | 1 | 1 |  |  | C/T | 0.211 | 0.207 | 0.004 |
| rs80226736 | 92921144 |  | 1 | 0 | 1 |  |  | T/C | 0.034 | 0.032 | 0.002 |
| rs79687662 | 92921291 |  | 1 | 0 | 1 |  |  | C/T | 0.044 | 0.052 | -0.007 |
| 15:92921534 | 92921534 | Y | ND | ND | 1 |  |  | A/G | 0.011 | 0 | 0.011 |
| 15:92921622 | 92921622 | Y | ND | ND | 1 |  |  | A/G | 0.011 | 0 | 0.011 |
| rs79935728 | 92921824 |  | 0 | 1 | 1 |  |  | A/G | 0.011 | 0.04 | -0.029 |
| rs117516664 | 92922095 |  | 0 | 1 | 1 |  |  | C/T | 0.054 | 0.052 | 0.003 |
| rs77271627 | 92922097 |  | 1 | 0 | 1 |  |  | C/T | 0.109 | 0.103 | 0.005 |
| **rs10852173** | **92922848** |  | **0** | **0** | **1** |  |  | **A/T** | **0.083** | **0.147** | **-0.063** |
| 15:92923096 | 92923096 | Y | 1 | 0 | 1 |  |  | G/A | 0.022 | 0 | 0.022# |
| rs12440999 | 92923152 |  | 1 | 0 | 1 |  |  | G/C | 0.033 | 0.035 | -0.001 |
| rs117300882 | 92923247 |  | 0 | 0 | 1 |  |  | T/C | 0.022 | 0.023 | -0.001 |
| rs190966523 | 92923469 |  | 0 | 0 | 1 |  |  | T/A | 0.011 | 0 | 0.011 |
| **rs56027313** | **92923947** |  | **0** | **0** | **1** |  |  | **G/C** | **0.061** | **0.138** | **-0.077** |
| rs7172479 | 92923990 |  | ND | ND | 3a |  |  | A/G | ND | ND | ND |
| rs55972842 | 92924086 |  | 0 | 0 | 1 |  |  | G/A | 0.077 | ND | ND |
| rs55905294 | 92924092 |  | 0 | 0 | 1 |  |  | A/G | 0.258 | ND | ND |
| rs113546420 | 92924095 |  | ND | ND | 3a |  |  |  | ND | 0 | ND |
| rs56016639 | 92924097 |  | 0 | 0 | 1 |  |  | G/A | 0.125 | 0.1149 | 0.0101 |
| rs56214704 | 92924112 |  | 1 | 0 | 1 |  |  | G/A | 0.111 | ND | ND |
| 15:92924364 | 92924364 | Y | ND | ND | 3b |  |  |  | ND | 0 | ND |
| rs28832394 | 92924449 |  | 1 | 0 | 1 |  |  | G/A | 0.033 | 0.035 | -0.002 |
| rs74758899 | 92925154 |  | 1 | 0 | 1 |  |  | C/G | 0.034 | 0.035 | 0 |
| rs145606077 | 92925177 |  | 0 | 0 | 1 |  |  | C/T | 0.011 | 0 | 0.011 |
| rs2200674 | 92926039 |  | 1 | 0 | 1 |  | 0.54 | C/A | 0.033 | 0.035 | -0.001 |
| 15:92926056 ^v^ | 92926056 | Y | 0 | 0 | 1 |  | 4.37 | A/G | 0.011 | 0 | 0.011 |
| rs2220386 | 92926118 |  | 1 | 0 | 1 |  |  | C/T | 0.011 | 0.035 | -0.023 |
| rs55649570 | 92926281 |  | 1 | 0 | 1 |  |  | G/C | 0.198 | 0.21 | -0.012 |
| **rs2129797** | **92926343** |  | **0** | **1** | **1** |  |  | **G/A** | **0.047** | **0.101** | **-0.054** |
| **rs2129796** | **92926357** |  | **0** | **1** | **1** |  |  | **A/G** | **0.047** | **0.101** | **-0.054** |
| rs6416575 | 92926535 |  | 0 | 0 | 1 |  | 3.2 |  |  | 0 | 0 |
| **rs12913269** | **92926705** |  | **0** | **1** | **1** |  |  | **T/C** | **0.034** | **0.101** | **-0.067** |
| **rs13379489** | **92926751** |  | **0** | **0** | **1** |  |  | **T/G** | **0.047** | **0.135** | **-0.089#** |
| rs17646351 | 92926831 |  | 1 | 0 | 1 |  |  | C/A | 0.012 | 0.035 | -0.023 |
| rs117156502 | 92926966 |  | 2 | 2 | 1 | 1,2 |  | T/C | 0.022 | 0.009 | 0.014 |
| rs28496195 | 92926968 |  | 1 | 0 | 1 | 1,2 |  | A/G | 0.011 | 0.035 | -0.023 |
| rs4777968 | 92927042 |  | 0 | 0 | 1 |  |  | G/A | 0.011 | 0.003 | 0.008 |
| rs111488721 | 92927202 |  | 0 | 0 | 1 |  |  | G/C | 0.068 | 0.06 | 0.008 |
| **rs16946839 ^v^** | **92928106** |  | **0** | **1** | **1** |  |  | **C/A** | **0.044** | **0.101** | **-0.056** |
| rs118099467 | 92928396 |  | 0 | 0 | 1 |  |  | A/T | 0.022 | 0.023 | -0.001 |
| **rs16946843** | **92928587** |  | **ND** | **ND** | **3a** | **2** |  | **T/C** | **ND** | **0.101** | **ND** |
| **rs7175280 ^v^** | **92928878** |  | **0** | **0** | **1** |  | 0.95 | **T/A** | **0.056** | **0.135** | **-0.08#** |
| **rs8035924 ^v^** | **92929144** |  | **0** | **1** | **1** |  |  | **G/T** | **0.044** | **0.101** | **-0.056** |
| 15:92929229 ^v^ | 92929229 | Y | 0 | 0 | 1 |  |  | C/G | 0.011 | 0 | 0.011 |
| rs76446952 | 92929404 |  | 0 | 0 | 1 |  |  | T/C | 0.054 | 0.046 | 0.008 |
| rs75043863 | 92929409 |  | 0 | 1 | 1 |  |  | A/G | 0.033 | 0.02 | 0.013 |
| rs4583192 ^v^ | 92929613 |  | ND | ND | 3b |  |  |  | ND | 0 | **ND** |
| rs8041550 ^v^ | 92930077 |  | 0 | 1 | 1 |  |  | T/C | 0.056 | 0.101 | -0.045 |
| rs8035799 | 92930293 |  | 0 | 0 | 1 |  |  |  |  | 0 | 0 |
| **rs12910290** | **92930408** |  | **0** | **1** | **1** |  |  | **A/G** | **0.043** | **0.101** | **-0.057** |
| rs17702901 | 92930411 |  | 1 | 0 | 1 |  |  | A/G | 0.011 | 0.035 | -0.024 |
| rs4570781 | 92930681 |  | 0 | 0 | 1 |  |  | C/T | 0.011 | 0 | 0.011 |
| rs4238483 | 92930785 |  | 0 | 0 | 1 |  |  |  |  | 0 | 0 |
| rs17646434 | 92931341 |  | 1 | 0 | 1 |  |  | T/G | 0.022 | 0.035 | -0.012 |
| **rs11630035** | **92931486** |  | **0** | **1** | **1** |  |  | **A/G** | **0.033** | **0.098** | **-0.065#** |
| rs17702960 | 92932529 |  | 1 | 0 | 1 |  |  | T/C | 0.011 | 0.035 | -0.023 |
| rs112425914 | 92932630 |  | 0 | 0 | 1 |  |  | G/C | 0.076 | 0.063 | 0.013 |
| rs142851738 | 92932659 |  | ND | ND | 3b |  |  |  | ND | 0 | ND |
| **rs7171140 ^v^** | **92932791** |  | **ND** | **ND** | **3a** |  | 2.78 | **A/G** | **ND** | **0.095** | **ND** |
| **rs7173401** | **92932887** |  | **ND** | **ND** | **3a** |  | -4.33 | **G/A** | **ND** | **0.098** | **ND** |
| rs6496930 | 92933262 |  | 0 | 0 | 1 |  |  |  | ND | 0 | ND |
| rs17646492 | 92933859 |  | 1 | 0 | 4 |  |  | A/G | 0.011 | 0.035 | -0.024 |
| rs8040424 ^v^ | 92934449 |  | 0 | 1 | 4 |  |  | A/C | 0.05435 | 0.0977 | -0.0434 |
| rs183300795 | 92934841 |  | ND | ND | 4 |  |  | C/T | 0.011 | 0.006 | 0.005 |
| rs186699149 | 92935000 |  | ND | ND | 4 | 2,3 |  |  | 0.01 | 0 | 0.01 |
| rs3759919 | 92935272 |  | 0 | 1 | 4 |  |  | A/T | 0.06383 | 0.0977 | -0.0339 |
| rs3759918 | 92935306 |  | 0 | 1 | 4 |  |  | C/A | 0.06383 | 0.0977 | -0.0339 |
| rs72766140 | 92935372 |  | 1 | 0 | 4 |  |  | T/C | 0.043 | 0.052 | -0.008 |
| rs3759917 | 92935447 |  | 0 | 0 | 4 |  |  | C/A | 0.09574 | 0.1322 | -0.0365 |
| rs3759916 | 92936169 |  | 0 | 0 | 4 |  | -1.83 |  | 0.01 | 0 | 0.01 |
| rs150882587 | 92936297 |  | ND | ND | 4 |  | -2.12 |  | 0.01 | 0 | 0.01 |
| 15:92936388 | 92936388 | Y | 1 | 0 | 4 |  |  |  | 0.031 | 0.043 | -0.012 |
| rs79668601 | 92936584 |  | 0 | 0 | 4 | 3 |  | A/G | 0.056 | 0.063 | -0.008 |
| rs138393846 | 92936774 |  | ND | ND | 4 | 2,3 | 4.28 |  | 0.01 | 0 | 0.01 |
| 15:92936875 | 92936875 | Y | ND | ND | 4 |  | 1.89 | G/T | 0.011 | 0 | 0.011 |
| **rs142883061*** | **92937137** |  | **0** | **1** | **4** | **1** | -2.84 | **C/T** | **0.053** | **0.106** | **-0.053** |
| **5’UTR & exon 1** |  |  |  |  |  |  |  |  |  |  |  |
| rs3743365* | 92937268 |  | 0 | 0 | 4 |  | 1.08 | G/T | 0.1064 | 0.1523 | -0.0459 |
| rs3743364* | 92937276 |  | 0 | 0 | 4 |  | 1.84 | C/T | 0.1064 | 0.1523 | -0.0459 |
| rs192224395 | 92937836 |  | ND | ND | 4 | 1,3 | 2.82 |  | 0.01 | 0 | 0.01 |
| 15:92937845 | 92937845 | Y | 2 | 2 | 4 | 1,3 | 4.12 | A/G | 0.011 | 0 | 0.011 |
| **rs3784745** | **92937858** |  | **0** | **0** | **4** | **1,2,3** | -0.45 | **G/C** | **0.0851** | **0.1437** | **-0.0586** |
| **rs3784744 ^v^** | **92937884** |  | **0** | **1** | **4** | **1,2,3** | 1.02 | **C/G** | **0.053** | **0.106** | **-0.053** |
| 15:92938316 | 92938316 | Y | ND | ND | 4 |  |  | G/C | 0.011 | 0 | 0.011 |
| rs7183703 | 92938342 |  | 0 | 1 | 4 |  |  | A/G | 0.065 | 0.078 | -0.012 |
| 15:92938435 | 92938435 | Y | ND | ND | 4 | 2 |  | A/G | 0.011 | 0 | 0.011 |
| rs73543829 | 92938826 |  | 0 | 0 | 1 | 3 |  | C/T | 0.011 | 0 | 0.011 |
| rs34744002 | 92939088 |  | 0 | 1 | 1 |  |  | T/G | 0.043 | 0.078 | -0.034 |
| rs12909593 | 92939770 |  | 1 | 0 | 1 |  |  | C/A | 0.054 | 0.081 | -0.026 |
| rs7174532 | 92939871 |  | 1 | 0 | 1 |  |  | C/G | 0.033 | 0.04 | -0.008 |
| rs79556496 | 92939872 |  | 1 | 0 | 1 |  |  | T/A | 0.033 | 0.04 | -0.008 |
| rs184091860 | 92939962 |  | 2 | 2 | 1 |  |  | A/G | 0.011 | 0 | 0.011 |
| rs11637377 | 92939998 |  | 0 | 1 | 1 | 3 |  | T/C | 0.022 | 0.055 | -0.032 |
| rs11857396 | 92940122 |  | 0 | 1 | 1 | 3 |  | A/G | 0.389 | 0.379 | 0.01 |
| rs4777969 ^v^ | 92940564 |  | 0 | 0 | 1 |  |  | A/G | 0.022 | 0.014 | 0.007 |
| **rs74029014 ^v^** | **92940731** |  | **0** | **0** | **1** |  |  | **G/A** | **0.022** | **0.083** | **-0.062#** |
| rs117727821 | 92940853 |  | 0 | 0 | 1 |  |  | T/C | 0.011 | 0.006 | 0.005 |
| rs8025225 | 92940900 |  | 0 | 1 | 1 |  |  | C/T | 0.446 | 0.448 | -0.003 |
| rs56254310 | 92941090 |  | 0 | 1 | 1 |  |  | T/C | 0.033 | 0.058 | -0.025 |
| rs11638862 | 92941160 |  | 0 | 1 | 1 |  |  | T/C | 0.413 | 0.391 | 0.022 |
| **rs6496931** | **92941343** |  | **1** | **0** | **1** |  |  | **A/G** | **0.5** | **0.44** | **0.06** |
| rs149543303 | 92941349 |  | 0 | 0 | 1 |  |  | C/G | 0.022 | 0.014 | 0.007 |
| rs117784318 | 92941559 |  | 0 | 1 | 1 |  |  | G/C | 0.022 | 0.012 | 0.01 |
| **rs2129795** | **92941980** |  | **0** | **0** | **1** |  |  | **A/G** | **0.033** | **0.092** | **-0.059#** |
| **rs2124359** | **92942089** |  | **0** | **0** | **1** |  |  | **G/C** | **0.054** | **0.106** | **-0.052#** |
| 15:92942136 | 92942136 | Y | 2 | 2 | 1 |  |  | C/A | 0.011 | 0 | 0.011 |
| **rs2124358** | **92942177** |  | **0** | **0** | **1** |  |  | **G/A** | **0.033** | **0.092** | **-0.059#** |
| rs2129794 | 92942306 |  | 0 | 0 | 1 |  |  | G/A | 0.022 | 0.017 | 0.005 |
| **rs2387806** | **92942367** |  | **0** | **0** | **1** |  |  | **G/A** | **0.033** | **0.092** | **-0.059#** |
| **rs2129793** | **92942575** |  | **0** | **0** | **1** |  |  | **A/C** | **0.033** | **0.092** | **-0.059#** |
| rs77748939 | 92942748 |  | 0 | 0 | 1 |  |  | A/G | 0.011 | 0.006 | 0.005 |
| rs142549699 | 92942785 |  | 2 | 2 | 1 |  |  | T/C | 0.011 | 0.003 | 0.008 |
| **rs722645 ^v^** | **92943323** |  | **0** | **1** | **1** | **3** |  | **G/A** | **0.489** | **0.563** | **-0.074** |
| rs28680784 | 92943349 |  | 0 | 1 | 1 | 3 |  | T/C | 0.424 | 0.391 | 0.033 |
| rs62020974 | 92943759 |  | 1 | 0 | 1 |  |  | A/C | 0.054 | 0.032 | 0.023 |
| **rs716628** | **92943773** |  | **0** | **0** | **1** |  |  | **A/G** | **0.022** | **0.083** | **-0.062#** |
| **rs921846 ^v^** | **92943966** |  | **1** | **0** | **1** | **2** |  | **A/G** | **0.489** | **0.555** | **-0.066** |
| rs67607760 | 92944461 |  | 1 | 0 | 1 |  |  | A/G | 0.065 | 0.055 | 0.011 |
| **rs72655696 ^v^** | **92944562** |  | **ND** | **ND** | **1** |  | -1.12 | **A/G** | **0.022** | **0.083** | **-0.062#** |
| **rs72655697** | **92944595** |  | **ND** | **ND** | **1** |  | 3.82 | **C/G** | **0.022** | **0.083** | **-0.062#** |
| rs143697694 ^v^ | 92944646 |  | 0 | 0 | 1 |  | 2.52 | G/A | 0.011 | 0.006 | 0.005 |
| rs11074064 ^v^ | 92944662 |  | 0 | 1 | 1 | 3 | 3.91 | A/G | 0.457 | 0.471 | -0.015 |
| **rs11074065** | **92944835** |  | **0** | **1** | **1** | **3** |  | **A/G** | **0.371** | **0.48** | **-0.109#** |
| **rs11074066 ^v^** | **92944864** |  | **0** | **1** | **1** |  |  | **A/C** | **0.556** | **0.463** | **0.093** |
| **rs1487984** | **92945117** |  | **0** | **0** | **1** |  |  | **T/G** | **0.015** | **0.086** | **-0.071#** |
| rs4777970 | 92945323 |  | 2 | 2 | 1 |  |  | A/G | 0.013 | 0 | 0.013 |
| **rs12592946** | **92945849** |  | **0** | **1** | **1** |  |  | **G/A** | **0.41** | **0.486** | **-0.075** |
| **rs12898703** | **92945975** |  | **0** | **1** | **1** |  |  | **T/C** | **0.575** | **0.457** | **0.118#** |
| **rs12593000** | **92946033** |  | **0** | **0** | **1** |  |  | **G/A** | **0.013** | **0.083** | **-0.071#** |
| rs79749850 | 92946101 |  | 0 | 0 | 1 |  |  | C/G | 0.038 | 0.017 | 0.021 |
| **rs11074067** | **92946298** |  | **0** | **1** | **1** |  |  | **C/G** | **0.413** | **0.486** | **-0.073** |
| rs2035645 | 92946326 |  | 0 | 1 | 1 |  |  | T/G | 0.513 | 0.56 | -0.048 |
| **rs73545807** | **92946973** |  | **0** | **0** | **1** |  |  | **T/C** | **0.013** | **0.083** | **-0.07#** |
| rs60862621 | 92946988 |  | 0 | 1 | 1 |  |  | G/C | 0.385 | 0.399 | -0.015 |
| rs72766144 | 92947401 |  | 1 | 0 | 1 |  |  | T/C | 0.125 | 0.092 | 0.033 |
| rs12442252 | 92947831 |  | 0 | 1 | 1 |  |  | T/C | 0.527 | 0.543 | -0.016 |
| rs4777971 | 92947868 |  | 0 | 1 | 1 |  |  | A/G | 0.527 | 0.535 | -0.007 |
| rs4777972 | 92948293 |  | 0 | 0 | 1 |  |  | G/A | 0.026 | 0.009 | 0.018 |
| rs11074068 | 92948584 |  | ND | ND | 3b |  |  |  | ND | 0.399 | ND |
| rs11074069 | 92948605 |  | 0 | 1 | 1 |  |  | G/T | 0.395 | 0.399 | -0.005 |
| rs147427286 | 92948645 |  | 0 | 1 | 1 |  |  | C/A | 0.039 | 0.02 | 0.019 |
| rs881770 | 92949809 |  | 0 | 1 | 1 |  |  | A/G | 0.513 | 0.546 | -0.033 |
| rs145860308 | 92950085 |  | 1 | 0 | 1 |  |  | A/G | 0.051 | 0.052 | 0 |
| rs60407423 | 92950096 |  | 0 | 1 | 1 |  |  | T/C | 0.423 | 0.391 | 0.032 |
| 15:92950518 | 92950518 | Y | 0 | 0 | 1 |  |  | C/T | 0.014 | 0 | 0.014 |
| rs4777973 | 92950883 |  | 0 | 1 | 1 |  |  | A/G | 0.449 | 0.414 | 0.035 |
| rs11853083 | 92951142 |  | 0 | 1 | 1 | 3 | 0.15 | G/A | 0.436 | 0.411 | 0.025 |
| rs17599821 | 92951358 |  | 0 | 1 | 1 | 2,3 |  | T/C | 0.41 | 0.408 | 0.002 |
| rs77386572 | 92951499 |  | 0 | 0 | 1 |  |  | A/G | 0.038 | 0.017 | 0.02 |
| **rs12438804** | **92951548** |  | **0** | **0** | **1** |  |  | **G/A** | **0.013** | **0.072** | **-0.059#** |
| 15:92951803 | 92951803 | Y | 0 | 0 | 1 |  |  | T/C | 0.013 | 0 | 0.013 |
| **rs57948373** | **92951907** |  | **0** | **0** | **1** |  |  | **A/T** | **0.154** | **0** | **0.154#** |
| rs62020978 | 92951975 |  | 0 | 1 | 1 |  |  | A/G | 0.397 | 0.402 | -0.005 |
| rs11636650 | 92952212 |  | 0 | 0 | 1 |  |  | C/G | 0.039 | 0.043 | -0.004 |
| rs4777974 ^v^ | 92952290 |  | 0 | 1 | 1 |  |  | G/A | 0.487 | 0.56 | **-0.074** |
| **rs7176813** | **92952633** |  | **0** | **0** | **1** |  | -3.86 | **A/G** | **0.077** | **0.175** | **-0.098#** |
| rs118032995 | 92952834 |  | 0 | 0 | 1 | 3 |  | T/A | 0.013 | 0.006 | 0.007 |
| rs11637898 | 92952850 |  | 0 | 1 | 1 | 3 |  | A/G | 0.438 | 0.434 | 0.004 |
| rs6496933 | 92953305 |  | 0 | 1 | 1 |  |  | G/A | 0.526 | 0.543 | -0.018 |
| rs4777975 | 92953726 |  | 0 | 1 | 1 |  |  | A/G | 0.438 | 0.428 | 0.009 |
| **rs4777976** | **92953730** |  | **0** | **1** | **1** |  |  | **T/C** | **0.475** | **0.552** | **-0.077** |
| rs138166632 | 92953965 |  | 2 | 2 | 1 |  |  | A/G | 0.013 | 0.006 | 0.007 |
| rs17599828 | 92953990 |  | 0 | 0 | 1 |  |  | G/A | 0.013 | 0.006 | 0.007 |
| rs10162611 | 92954316 |  | 0 | 1 | 1 |  |  | A/G | 0.423 | 0.434 | -0.011 |
| rs3887775 | 92955017 |  | 0 | 1 | 1 |  |  | C/T | 0.434 | 0.443 | -0.008 |
| rs148606815 | 92955426 |  | 0 | 0 | 1 |  |  | C/G | 0.013 | 0.006 | 0.007 |
| rs78286452 | 92955528 |  | 0 | 0 | 1 |  |  | G/A | 0.013 | 0.009 | 0.004 |
| rs118081684 | 92955639 |  | ND | ND | 3a |  |  | G/A | ND | 0.006 | ND |
| 15:92956314 ^v^ | 92956314 | Y | 0 | 1 | 1 |  |  | G/A | 0.026 | 0 | 0.026# |
| rs8026158 | 92956373 |  | 0 | 1 | 1 |  |  | A/C | 0.423 | 0.411 | 0.012 |
| rs17599870 | 92957744 |  | 0 | 0 | 1 |  |  | G/C | 0.011 | 0.006 | 0.005 |
| rs4777977 | 92957793 |  | 0 | 1 | 1 |  |  | A/G | 0.413 | 0.434 | -0.021 |
| rs3825986 | 92958095 |  | 0 | 1 | 1 |  |  | A/G | 0.435 | 0.431 | 0.004 |
| 15:92958227 ^v^ | 92958227 | Y | ND | ND | 1 |  |  | G/C | 0.033 | 0 | 0.033# |
| 15:92958793 ^v^ | 92958793 | Y | 0 | 0 | 1 |  |  | A/C | 0.011 | 0 | 0.011 |
| rs147515876 | 92959331 |  | 0 | 0 | 1 |  |  | G/A | 0.011 | 0.003 | 0.008 |
| 15:92959424 ^v^ | 92959424 | Y | 2 | 2 | 2 |  |  | G/A | 0.011 | 0 | 0.011 |
| rs116924409 | 92960116 |  | 0 | 0 | 1 |  |  | A/G | 0.011 | 0.006 | 0.005 |
| rs17521544 | 92960137 |  | 0 | 0 | 1 |  |  | T/C | 0.011 | 0.009 | 0.002 |
| rs3784740 | 92960475 |  | 0 | 1 | 1 |  |  | G/A | 0.391 | 0.425 | -0.034 |
| rs3848152 | 92960997 |  | 0 | 1 | 1 |  |  | A/G | 0.411 | 0.414 | -0.003 |
| rs3848153 | 92961012 |  | 0 | 1 | 1 |  |  | T/C | 0.4 | 0.405 | -0.005 |
| 15:92961050 ^v^ | 92961050 | Y | ND | ND | 3b |  |  | A/G | ND | 0 | ND |
| rs3858915 | 92961234 |  | 0 | 1 | 1 |  |  | T/G | 0.409 | 0.405 | 0.004 |
| 15:92961385 ^v^ | 92961385 | Y | 2 | 2 | 1 |  |  | G/A | 0.011 | 0 | 0.011 |
| rs12592754 | 92961617 |  | 0 | 0 | 1 |  |  | A/G | 0.452 | 0.425 | 0.027 |
| rs137875787 | 92961625 |  | 0 | 0 | 1 |  |  | A/G | 0.012 | 0.006 | 0.006 |
| rs12592896 | 92961670 |  | 0 | 0 | 1 |  |  | T/C | 0.45 | 0.425 | 0.025 |
| rs12592918 | 92961688 |  | 2 | 2 | 1 |  |  | C/A | 0.013 | 0 | 0.013 |
| 15:92962229 | 92962229 | Y | 0 | 0 | 3a | 1,2 |  | C/T | ND | 0 | ND |
| rs112592012 | 92962430 |  | 0 | 0 | 1 |  |  | A/G | 0.011 | 0.014 | -0.003 |
| rs4777978 | 92962762 |  | ND | ND | 3b |  |  |  | ND | 0.431 | ND |
| 15:92962920 | 92962920 | Y | 0 | 0 | 1 |  |  | G/T | 0.011 | 0 | 0.011 |
| rs4777710 | 92963111 |  | 0 | 0 | 1 |  |  | G/C | 0.422 | 0.437 | -0.015 |
| rs3848154 | 92963285 |  | 0 | 0 | 1 |  |  | T/C | 0.411 | 0.425 | -0.014 |
| rs113984989 | 92963546 |  | ND | ND | 3a |  |  | C/T | ND | 0.006 | ND |
| rs189943926 | 92963849 |  | 0 | 0 | 1 |  | 0.91 | G/A | 0.011 | 0 | 0.011 |
| rs3784738 | 92963857 |  | 0 | 0 | 1 |  | 2.02 | A/G | 0.446 | 0.428 | 0.018 |
| rs112750488 | 92964307 |  | 0 | 0 | 1 |  |  | T/C | 0.011 | 0.003 | 0.008 |
| rs16946889 | 92964428 |  | 0 | 0 | 1 |  |  | C/G | 0.011 | 0.009 | 0.002 |
| rs4777979 | 92965421 |  | 0 | 1 | 1 |  |  | A/G | 0.433 | 0.411 | 0.022 |
| rs4777980 | 92965577 |  | 0 | 1 | 1 |  |  | A/G | 0.398 | 0.417 | -0.019 |
| rs11074070 | 92966826 |  | 0 | 1 | 1 |  |  | G/T | 0.409 | 0.425 | -0.016 |
| 15:92967421 | 92967421 | Y | 0 | 0 | 1 |  | 0.68 | C/T | 0.022 | 0 | 0.022# |
| rs3784737 | 92967552 |  | 0 | 1 | 1 |  |  | C/T | 0.367 | 0.342 | 0.025 |
| rs11635453 | 92968587 |  | 0 | 1 | 1 |  |  | A/G | 0.421 | 0.425 | -0.005 |
| 15:92969392 | 92969392 | Y | ND | ND | 3b | 3 |  |  | ND | 0 | **ND** |
| rs72766148 | 92969665 |  | 2 | 2 | 1 |  |  | T/A | 0.011 | 0.012 | -0.001 |
| rs79135429 | 92969886 |  | 0 | 0 | 1 |  |  | C/G | 0.011 | 0.006 | 0.005 |
| rs2892364 | 92970905 |  | 0 | 1 | 1 |  |  | C/T | 0.424 | 0.44 | -0.016 |
| rs76589571 | 92971389 |  | 0 | 1 | 1 |  |  | T/A | 0.043 | 0.02 | 0.023 |
| rs3858917 | 92971811 |  | 0 | 1 | 1 |  |  | G/T | 0.424 | 0.417 | 0.007 |
| rs3825985 | 92972302 |  | 0 | 1 | 1 |  |  | A/G | 0.391 | 0.425 | -0.034 |
| rs72766149 | 92972306 |  | 1 | 0 | 1 |  |  | T/C | 0.109 | 0.095 | 0.014 |
| rs17600009 | 92972782 |  | 0 | 0 | 1 |  |  | G/A | 0.011 | 0.006 | 0.005 |
| Exon 2 | | | | | | | | | | | |
| 15:92973414 ^v^ | 92973414 | Y | 2 | 2 | 1 | 1 | 5.55 | A/G | 0.011 | 0 | 0.011 |
| rs12910599 | 92973965 |  | 1 | 0 | 1 |  |  | A/G | 0.087 | 0.098 | -0.011 |
| rs118049346 | 92974353 |  | 0 | 0 | 1 |  |  | T/C | 0.033 | 0.02 | 0.013 |
| rs8037133 | 92974457 |  | 0 | 0 | 1 |  |  | A/G | 0.446 | 0.437 | 0.009 |
| 15:92974592 | 92974592 | Y | ND | ND | 3a |  |  | G/C | ND | 0 | ND |
| rs143694150 | 92974595 |  | ND | ND | 3a |  |  | T/A | ND | 0.006 | ND |
| rs3931230 | 92974636 |  | 0 | 0 | 1 |  |  | A/C | 0.435 | 0.437 | -0.002 |
| rs3784736 | 92974981 |  | ND | ND | 3b |  |  |  | ND | 0.448 | ND |
| rs75902835 | 92975005 |  | 0 | 0 | 1 |  |  | C/G | 0.011 | 0.017 | -0.006 |
| rs11857313 | 92975271 |  | 0 | 0 | 1 |  |  | T/C | 0.011 | 0.006 | 0.005 |
| rs3784735 | 92975329 |  | 0 | 1 | 3a |  |  | C/A | ND | 0.431 | ND |
| rs3825984 | 92975435 |  | 0 | 0 | 1 |  |  | G/A | 0.457 | 0.443 | 0.014 |
| rs3848155 | 92976045 |  | 0 | 1 | 1 |  |  | A/G | 0.424 | 0.44 | -0.016 |
| rs3848156 | 92976194 |  | 0 | 0 | 1 |  |  | C/T | 0.457 | 0.448 | 0.008 |
| rs193188732 | 92976405 |  | 0 | 0 | 1 |  |  | T/A | 0.011 | 0.009 | 0.002 |
| rs3902203 | 92976599 |  | 0 | 0 | 1 |  |  | G/A | 0.457 | 0.448 | 0.008 |
| rs1869777 | 92977098 |  | 0 | 0 | 1 |  |  | C/A | 0.446 | 0.448 | -0.003 |
| Exon 3 | | | | | | | | | | | |
| rs2242113 | 92977670 |  | 0 | 1 | 1 |  |  | G/T | 0.435 | 0.434 | 0.001 |
| rs8035191 | 92978023 |  | 0 | 0 | 1 |  |  | C/T | 0.457 | 0.491 | -0.035 |
| rs76243729 | 92978149 |  | ND | ND | 3b |  |  |  | ND | 0 | ND |
| **rs35848755** | **92978151** |  | **0** | **1** | **2** |  |  | **A/G** | **0.446** | **0** | **0.446#** |
| rs7174021 | 92978788 |  | ND | ND | 3a |  |  | A/G | ND | 0.399 | ND |
| rs7180785 | 92978789 |  | ND | ND | 3a |  |  | C/T | ND | 0.411 | ND |
| rs146634540 | 92979109 |  | 2 | 2 | 1 |  |  | T/C | 0.011 | 0.009 | 0.002 |
| rs7176692 | 92979163 |  | 0 | 0 | 1 |  |  | G/A | 0.011 | 0.012 | -0.001 |
| rs76415114 | 92979173 |  | ND | ND | 3b |  |  |  | ND | 0.009 | ND |
| rs56855156 | 92979200 |  | 1 | 0 | 1 |  |  | A/C | 0.261 | 0.216 | 0.045 |
| rs11074071 | 92979209 |  | ND | ND | 3a |  |  | G/A | ND | 0.463 | ND |
| rs7177050 | 92979333 |  | 0 | 0 | 1 |  |  | G/A | 0.446 | 0.463 | -0.017 |
| rs7181934 | 92979398 |  | ND | ND | 3b |  |  |  | ND | 0.009 | ND |
| 15:92979413 | 92979413 | Y | 0 | 0 | 1 |  |  | A/G | 0.011 | 0 | 0.011 |
| rs75981356 | 92979440 |  | 0 | 0 | 1 |  |  | C/G | 0.011 | 0.009 | 0.002 |
| rs11632521 | 92979447 |  | 0 | 1 | 1 |  |  | A/G | 0.435 | 0.443 | -0.008 |
| **rs111353089** | **92979525** |  | **0** | **1** | **1** |  |  | **G/A** | **0.065** | **0.012** | **0.054#** |
| rs79332614 | 92979561 |  | 1 | 0 | 1 |  |  | A/G | 0.022 | 0.02 | 0.002 |
| rs138941579 | 92979979 |  | 2 | 2 | 1 |  |  | G/A | 0.011 | 0.009 | 0.002 |
| rs11634070 | 92980046 |  | 0 | 1 | 1 |  |  | A/C | 0.065 | 0.103 | -0.038 |
| rs60704830 | 92980202 |  | 0 | 0 | 1 |  |  | C/T | 0.011 | 0.009 | 0.002 |
| rs17521726 | 92980544 |  | ND | ND | 3a |  |  | T/G | ND | 0.443 | ND |
| rs78382491 | 92980734 |  | 0 | 0 | 1 |  |  | A/G | 0.022 | 0.02 | 0.002 |
| rs7166312 | 92980784 |  | ND | ND | 3a |  |  | G/A | ND | 0.012 | ND |
| rs7166337 | 92980845 |  | 0 | 1 | 1 |  |  | T/A | 0.402 | 0.451 | -0.049 |
| rs9920408 | 92980869 |  | 0 | 1 | 1 |  |  | C/T | 0.402 | 0.443 | -0.04 |
| rs17521740 | 92980967 |  | 0 | 1 | 1 |  |  | A/G | 0.402 | 0.443 | -0.04 |
| rs4777981 | 92981064 |  | 0 | 1 | 1 |  |  | A/G | 0.402 | 0.437 | -0.035 |
| rs4777982 | 92981194 |  | 0 | 1 | 1 |  |  | G/T | 0.402 | 0.44 | -0.038 |
| rs12438164 | 92981359 |  | 0 | 1 | 1 |  |  | C/A | 0.413 | 0.443 | -0.03 |
| rs17521754 | 92981390 |  | 0 | 1 | 1 |  |  | A/G | 0.413 | 0.443 | -0.03 |
| rs1017672 | 92981424 |  | 0 | 0 | 1 |  |  | C/G | 0.413 | 0.463 | -0.05 |
| Exon 4 | | | | | | | | | | | |
| rs112804054 | 92981903 |  | 2 | 2 | 1 | 1 |  | C/A | 0.011 | 0.009 | 0.002 |
| rs12441748 | 92982076 |  | 0 | 1 | 1 |  |  | A/G | 0.348 | 0.348 | 0 |
| rs12441750 | 92982096 |  | 0 | 0 | 1 |  |  | T/G | 0.359 | 0.356 | 0.002 |
| rs4777711 | 92982265 |  | 0 | 0 | 1 |  |  | G/A | 0.413 | 0.451 | -0.038 |
| rs4777983 | 92982454 |  | ND | ND | 3a |  |  | A/G | ND | 0 | ND |
| rs4777712 | 92982649 |  | 0 | 0 | 1 |  | 1.07 | G/A | 0.413 | 0.451 | -0.038 |
| rs148555352 | 92983044 |  | 0 | 1 | 1 |  |  | A/G | 0.043 | 0.012 | 0.032 |
| rs2168351 | 92983722 |  | 0 | 0 | 1 |  |  | G/A | 0.359 | 0.348 | 0.011 |
| rs6496938 | 92983850 |  | 0 | 0 | 1 |  |  | A/T | 0.435 | 0.468 | -0.034 |
| rs7183904 | 92984203 |  | 0 | 0 | 1 |  |  | C/G | 0.37 | 0.345 | 0.025 |
| rs11074072 | 92984506 |  | 0 | 0 | 1 |  |  | A/G | 0.435 | 0.466 | -0.031 |
| 15:92984673 | 92984673 | Y | 0 | 0 | 1 | 2 |  | A/G | 0.011 | 0 | 0.011 |
| 15:92984841 | 92984841 | Y | ND | ND | 3b |  |  |  | ND | 0 | ND |
| rs1455780 | 92985006 |  | 0 | 0 | 1 |  |  | A/G | 0.446 | 0.471 | -0.026 |
| rs79234879 | 92985021 |  | 0 | 0 | 1 |  |  | A/G | 0.011 | 0.009 | 0.002 |
| rs8024780 | 92985689 |  | 0 | 0 | 1 |  |  | G/C | 0.391 | 0.431 | -0.04 |
| rs8030186 | 92985882 |  | 0 | 0 | 1 |  |  | A/T | 0.283 | 0.299 | -0.016 |
| 15:92985963 | 92985963 | Y | 2 | 2 | 1 |  |  | C/G | 0.011 | 0 | 0.011 |
| rs6496939 | 92986363 |  | 0 | 0 | 1 |  |  | G/A | 0.294 | 0.31 | -0.017 |
| rs3784734 | 92986861 |  | ND | ND | 3a |  |  | C/T | ND | 0.445 | ND |
| rs3784733 | 92986870 |  | ND | ND | 3a |  |  | C/A | ND | 0.313 | ND |
| rs3784732 | 92986916 |  | 0 | 0 | 1 |  |  | G/A | 0.087 | 0.121 | -0.034 |
| rs3784731 | 92987079 |  | 0 | 0 | 1 |  |  | T/A | 0.422 | 0.454 | -0.032 |
| rs3784730 ^v^ | 92987128 |  | 0 | 0 | 1 |  |  | G/A | 0.456 | 0.428 | 0.027 |
| rs74029037 | 92987146 |  | 0 | 1 | 1 |  |  | T/C | 0.011 | 0.032 | -0.02 |
| 15:92987199 | 92987199 | Y | ND | ND | 3a |  |  |  | ND | 0 | ND |
| rs1869775 | 92987563 |  | 0 | 0 | 1 |  |  | C/T | 0.089 | 0.132 | -0.043 |
| **Exon 5** |  |  |  |  |  |  |  |  |  |  |  |
| rs2305561* | 92987938 |  | 0 | 0 | 1 |  | -0.19 | G/C | 0.1 | 0.115 | -0.015 |
| rs11637874 | 92988165 |  | 0 | 0 | 1 |  |  | T/C | 0.114 | 0.118 | -0.004 |
| **rs62021045 ^v^** | **92988391** |  | **0** | **0** | **1** |  |  | **A/G** | **0.489** | **0.408** | **0.081#** |
| **rs62021046** | **92988476** |  | **0** | **0** | **1** |  |  | **T/C** | **0.489** | **0.408** | **0.081#** |
| rs1455779 | 92988649 |  | 0 | 0 | 1 |  |  | C/T | 0.435 | 0.46 | -0.025 |
| rs1455778 | 92988659 |  | 0 | 0 | 1 |  |  | G/C | 0.424 | 0.457 | -0.033 |
| rs1455777 | 92988741 |  | 0 | 0 | 1 |  |  | A/G | 0.413 | 0.46 | -0.047 |
| 15:92988931 | 92988931 | Y | ND | ND | 3b |  |  |  | ND | 0 | ND |
| rs2045269 | 92989472 |  | 0 | 0 | 1 |  |  | T/C | 0.444 | 0.408 | 0.036 |
| rs2045268 | 92989647 |  | 0 | 0 | 1 |  |  | T/C | 0.433 | 0.402 | 0.031 |
| rs28668996 | 92990010 |  | 1 | 0 | 1 |  |  | T/C | 0.067 | 0.095 | -0.028 |
| rs11630692 | 92990153 |  | 0 | 0 | 1 |  |  | T/C | 0.089 | 0.118 | -0.029 |
| rs62021047 | 92990324 |  | 0 | 1 | 1 |  |  | A/G | 0.067 | 0.046 | 0.021 |
| rs7166344 | 92990342 |  | 0 | 0 | 1 |  |  | G/A | 0.422 | 0.451 | -0.029 |
| rs139293010 | 92990793 |  | 0 | 1 | 1 |  |  | T/G | 0.022 | 0.014 | 0.007# |
| 15:92992055 ^v^ | 92992055 | Y | 0 | 1 | 1 |  |  | T/C | 0.022 | 0 | 0.022# |
| rs4777713 ^v^ | 92992642 |  | 0 | 0 | 1 |  |  | T/G | 0.411 | 0.454 | -0.043 |
| rs4777714 | 92992678 |  | 1 | 0 | 1 |  |  | A/G | 0.067 | 0.095 | -0.028 |
| rs62021048 | 92992965 |  | 1 | 0 | 1 |  |  | T/C | 0.08 | 0.037 | 0.042 |
| **rs12148510 ^v^** | **92993004** |  | **0** | **0** | **1** |  |  | **A/G** | **0.5** | **0.417** | **0.083#** |
| 15:92993375 ^v^ | 92993375 | Y | 0 | 0 | 1 |  |  | T/C | 0.011 | 0 | 0.011 |
| **rs56152327** | **92993468** |  | **0** | **0** | **1** |  |  | **G/C** | **0.5** | **0.44** | **0.06** |
| 15:92994038 ^v^ | 92994038 | Y | ND | ND | 3b |  |  |  | ND | 0 | **ND** |
| rs8032091 | 92994407 |  | 1 | 0 | 1 |  |  | G/A | 0.207 | 0.172 | 0.034 |
| 15:92994449 ^v^ | 92994449 | Y | 2 | 2 | 1 |  |  | A/G | 0.011 | 0 | 0.011 |
| rs1487982 | 92994912 |  | 1 | 0 | 1 |  |  | G/A | 0.159 | 0.198 | -0.039 |
| rs1352323 | 92995641 |  | 0 | 0 | 1 |  |  | A/G | 0.244 | 0.25 | -0.006 |
| rs3784729 | 92996007 |  | 0 | 0 | 1 |  |  | A/G | 0.261 | 0.256 | 0.006 |
| rs17521932 | 92996375 |  | 0 | 0 | 1 |  |  | C/T | 0.011 | 0.003 | 0.008 |
| rs12912127 ^v^ | 92996591 |  | 0 | 0 | 1 |  |  | A/C | 0.444 | 0.471 | -0.027 |
| rs10775256 | 92997155 |  | 0 | 0 | 1 |  |  | G/A | 0.352 | 0.342 | 0.01 |
| rs4777985 | 92997507 |  | ND | ND | 3b |  |  |  | ND | 0.328 | ND |
| rs28685646 | 92997632 |  | 0 | 0 | 1 |  |  | A/T | 0.011 | 0.003 | 0.008 |
| rs59585859 | 92998684 |  | ND | ND | 3b | 3 |  |  | ND | 0.135 | ND |
| rs6496942 | 92999093 |  | 0 | 1 | 1 |  |  | C/T | 0.044 | 0.055 | -0.01 |
| rs3784727 | 92999337 |  | 0 | 0 | 1 |  |  | C/G | 0.424 | 0.397 | 0.027 |
| rs75520114 | 92999373 |  | 0 | 1 | 1 |  |  | A/G | 0.043 | 0.055 | -0.011 |
| rs113803966 | 92999401 |  | 0 | 1 | 1 |  |  | T/G | 0.043 | 0.055 | -0.011 |
| rs74482817 | 92999496 |  | 1 | 0 | 1 |  |  | A/G | 0.043 | 0.058 | -0.014 |
| rs6496943 | 92999548 |  | 0 | 1 | 1 |  |  | C/A | 0.054 | 0.063 | -0.009 |
| rs59673848 | 93000081 |  | 0 | 0 | 1 |  |  | T/G | 0.098 | 0.135 | -0.037 |
| **rs76405928** | **93000441** |  | **0** | **0** | **1** | **3** |  | **T/A** | **0.457** | **0.379** | **0.077#** |
| rs79846035 | 93000995 |  | 1 | 0 | 1 | 3 | -2.22 | A/G | 0.054 | 0.049 | 0.005 |
| rs74895969 | 93001035 |  | 0 | 1 | 1 | 3 |  | A/G | 0.043 | 0.055 | -0.011 |
| rs74521141 | 93001329 |  | 0 | 1 | 1 |  |  | C/T | 0.043 | 0.055 | -0.011 |
| **rs11852344 ^v^** | **93001521** |  | **0** | **0** | **1** |  |  | **A/G** | **0.141** | **0.201** | **-0.06** |
| rs79173483 | 93001657 |  | 0 | 1 | 1 |  |  | A/G | 0.043 | 0.055 | -0.011 |
| rs117645273 | 93001800 |  | 0 | 0 | 1 |  |  | A/G | 0.011 | 0.02 | -0.009 |
| rs4777715 | 93002081 |  | 0 | 0 | 1 |  |  | G/A | 0.359 | 0.371 | -0.012 |
| rs4777987 | 93002182 |  | 0 | 0 | 1 |  |  | T/C | 0.37 | 0.376 | -0.007 |
| rs141457407 | 93002376 |  | 0 | 1 | 1 |  |  | C/T | 0.011 | 0.006 | 0.005 |
| rs4777988 | 93002444 |  | 0 | 0 | 1 | 2 |  | G/A | 0.424 | 0.431 | -0.007 |
| rs16946953 | 93002606 |  | 0 | 0 | 1 |  |  | C/G | 0.011 | 0 | 0.011 |
| 15:93002872 | 93002872 | Y | 0 | 0 | 1 |  |  | T/C | 0.011 | 0 | 0.011 |
| rs182345701 | 93003222 |  | 0 | 1 | 1 |  |  | T/C | 0.011 | 0 | 0.011 |
| rs3784726 | 93004267 |  | 0 | 0 | 1 |  |  | C/A | 0.043 | 0.078 | -0.034 |
| rs3825983 | 93004676 |  | 0 | 0 | 1 |  |  | A/G | 0.044 | 0.078 | -0.033 |
| rs3784724 | 93004694 |  | 0 | 0 | 1 |  |  | G/C | 0.044 | 0.078 | -0.033 |
| rs75892170 | 93004740 |  | 0 | 0 | 1 |  |  | T/C | 0.011 | 0.009 | 0.002 |
| rs189590347 | 93005437 |  | 0 | 0 | 1 |  |  | C/T | 0.011 | 0 | 0.011 |
| rs3784723 | 93005759 |  | 0 | 0 | 1 |  |  | G/A | 0.043 | 0.078 | -0.034 |
| **rs3784722** | **93006088** |  | **0** | **0** | **1** |  |  | **G/A** | **0.294** | **0.371** | **-0.077#** |
| rs3784721 | 93006509 |  | 0 | 0 | 1 |  |  | C/A | 0.057 | 0.078 | -0.021 |
| rs4777989 | 93006740 |  | 0 | 0 | 1 |  |  | G/A | 0.37 | 0.342 | 0.028 |
| **rs72655696** | **93006765** |  | **0** | **0** | **1** |  |  | **A/G** | **0.022** | **0.098** | **-0.076#** |
| rs62021050 | 93006888 |  | 0 | 0 | 1 |  |  | G/A | 0.217 | 0.181 | 0.036 |
| rs11629679 | 93006932 |  | 0 | 0 | 1 |  |  | A/C | 0.304 | 0.322 | -0.018 |
| **rs7168443 ^v^** | **93007049** |  | **0** | **0** | **1** |  |  | **T/C** | **0.315** | **0.233** | **0.082#** |
| **Exon 6 &** **3’UTR** |  |  |  |  |  |  |  |  |  |  |  |
| rs139149207* ^v^ | 93007785 |  | 0 | 0 | 1 |  | 5.45 | A/C | 0.011 | 0.003 | 0.008 |
| **rs2290492* ^v^** | **93007974** |  | **0** | **0** | **1** |  | 4.64 | **A/G** | **0.278** | **0.221** | **0.057** |
| rs12904773* | 93008168 |  | 0 | 1 | 1 |  |  | G/C | 0.065 | 0.037 | 0.028 |
| **rs8035760* ^v^** | **93008472** |  | **0** | **0** | **1** |  |  | **A/T** | **0.359** | **0.19** | **0.169#** |
| **rs1869774* ^v^** | **93010177** |  | **0** | **0** | **1** | **2** |  | **T/C** | **0.315** | **0.233** | **0.082#** |
| rs115781738* | 93010192 |  | 2 | 2 | 1 | 2 |  | C/G | 0.011 | 0 | 0.011 |
| rs116928729* | 93010321 |  | 0 | 0 | 1 |  |  | A/G | 0.022 | 0.017 | 0.005 |
| rs17600420* | 93011135 |  | 0 | 0 | 1 |  |  | G/A | 0.304 | 0.345 | -0.041 |
| rs117763930* | 93011237 |  | 0 | 0 | 1 |  |  | A/G | 0.022 | 0.017 | 0.005 |
| rs145948851* | 93011468 |  | 2 | 2 | 1 |  |  | C/T | 0.011 | 0 | 0.011 |
| **rs11853992 ^v^** | **93012351** |  | **0** | **0** | **1** | **3** | 2.51 | **G/A** | **0.33** | **0.2787** | **0.0513** |
| rs147920939 | 93012426 |  | 0 | 0 | 1 | 3 | -1.12 | A/G | 0.011 | 0.0086 | 0.0024 |
| rs17522085 | 93012557 |  | 0 | 0 | 1 |  | 3 | T/C | 0.213 | 0.1839 | 0.0291 |
| **rs12592811** | **93012620** |  | **0** | **0** | **1** |  |  | **G/A** | **0.287** | **0.3592** | **-0.0722** |
| rs11074073 | 93012627 |  | 0 | 0 | 1 |  |  | G/C | 0.309 | 0.2759 | 0.0331 |
| rs11634493 | 93013095 |  | 0 | 0 | 1 |  |  | T/C | 0.266 | 0.236 | 0.03 |
| rs62021051 | 93013194 |  | 0 | 0 | 1 |  |  | T/C | 0.074 | 0.0747 | -0.0007 |
| 15:93013520 | 93013520 | Y | 0 | 0 | 1 |  |  | A/C | 0.011 | 0 | 0.011 |
| rs2279447 | 93013560 |  | 0 | 0 | 1 |  |  | G/A | 0.309 | 0.3506 | -0.0416 |

The location of all identified variants on chromosome 15 *ST8SIA2 region* (hg19 build), with transcribed SNPs indicated with asterisks. SNPs identified exclusively on the risk haplotype are shown (0 = not exclusive, 1 = present on risk and other haplotypes, 2 = present on risk haplotype only, ND = not determined). SNPs identified exclusively on the protective haplotype are shown (0 = not exclusive, 1 = present on protective and other haplotypes, 2 = present on protective haplotype only, ND=not determined). The set from which the SNP was observed is given (1 = GATK & Refmapper; 2 = GATK only; 3a = Refmapper only; 3b = Refmapper only & filtered in GATK; 4 = Sanger). Co-localisation with DNase I hypersensitivity site peaks (DHSPs; neuronal=1; hESC=2; foetal brain=3) are given. Genomic Evolutionary Rate Profiling (GERP) scores are provided for each variant that is within a GERP-conserved element. The nature of the polymorphism in each cohort is given (with minor allele listed first). The minor allele frequency (mAF) of each variant in the 47 bipolar cases and 174 Caucasian individuals (CEU and GBR) from the 1000 Genomes Project (1kG) are shown separately. The frequency difference (freqDIFF) was calculated relative to 1kG allele frequency, and those with p values < 0.1 indicated with an asterisk. ^a^For each polymorphism, the minor allele is listed first. ^v^ SNPs verified by direct genotyping are indicated.
