# Supplementary material for: Characterisation of Genetic Variation in ST8SIA2 and Its Interaction Region in NCAM1 in Patients with Bipolar Disorder
Source: PLoS One. 2014 Mar 20;9(3):e92556. doi: 10.1371/journal.pone.0092556 (PMC3961385; doi:10.1371/journal.pone.0092556)
Supplement: Note S1 — GATK SNP Filtering Threshold Note S1: GATK SNP Filtering Threshold. (DOCX) [file pone.0092556.s004.docx]

“Characterisation of genetic variation in *ST8SIA2* and its interaction region in NCAM1 in patients with bipolar disorder”

**AD Shaw, Y Tiwari, W Kaplan, A Heath, PB Mitchell, PR Schofield, JM Fullerton**

## Supplementary Note 1: GATK SNP Filtering Threshold

Initially we used the GATK recommended filtering for genome wide sequencing (see methods). We inspected the SNPs that passed filtering, examining their reported allele frequency and quality scores in our data. We identified a subset of variants that passed filtering but were not reported in dbSNP 135, despite being at apparently high allele frequency in the bipolar cohort (MAF range=0.12-0.34; n=16; Figure S1). Given that genome‑wide variation has been thoroughly characterised in the Caucasian European population from which our individuals were drawn, and given the relatively low quality scores for these variants (QD < 5), it seemed unlikely that these were true novel variants.

On inspecting the alignment of raw sequence data at these sites, we found that all of these ‘novel’ sites with high allele frequency were within homopolymer-rich sequence contexts. Spurious ‘dinucleotide’ SNPs are known to arise next to homopolymers due to a ‘loss of synchrony’ in 454‑sequencing [1], whereby a false inserted base appears in the previous or next flow cycle relative to the homopolymer. Indeed all of the suspect calls with high allele frequency from our dataset were due to an inserted base with the same base identity as a proximal homopolymer, and are thus attributable to loss of synchrony artefacts. We confirmed by restriction digest one of these apparent common novel variants, which lay downstream of a homopolymer immediately adjacent to exon 6 (at 93,007,325 bp), was indeed a sequencing artefact (data not shown). As these calls had relatively low QD scores, we found that increasing the Quality By Depth (QD) threshold for filtering to the more stringent QD < 5 from the GATK recommended QD < 2 eliminated all of these sites, and resulted in 349 SNPs which passed filtering.

Although it is unlikely that all spurious calls could be removed using a filtering-by-threshold approach, and conversely that all genuine SNPs will pass filtering, increasing the stringency from recommended genome-wide filtering in this manner resulted in: i) a Ti/Tv ratio of 2.26, which is just outside the expectation for whole-genome sequence data [2.0-2.2; 2,3]; ii) a similar proportion of novel calls/total calls to an independent algorithm (Table 1), and iii) a high rate of accuracy of diploid genotype calling at SNP sites that passed filtering (Table 2).

**References**

1. Hoberman R, Dias J, Ge B, Harmsen E (2009) A probabilistic approach for SNP discovery in high-throughput human resequencing data. Genome Res: 1542–1552. doi:10.1101/gr.092072.109.on.

2. DePristo M a, Banks E, Poplin R, Garimella K V, Maguire JR, et al. (2011) A framework for variation discovery and genotyping using next-generation DNA sequencing data. Nat Genet 43: 491–498. doi:10.1038/ng.806.

3. Freudenberg-Hua Y, Freudenberg J, Kluck N, Cichon S, Propping P, et al. (2003) Single nucleotide variation analysis in 65 candidate genes for CNS disorders in a representative sample of the European population. Genome Res 13: 2271–2276. doi:10.1101/gr.1299703.
