# Supplementary material for: Characterisation of Genetic Variation in ST8SIA2 and Its Interaction Region in NCAM1 in Patients with Bipolar Disorder
Source: PLoS One. 2014 Mar 20;9(3):e92556. doi: 10.1371/journal.pone.0092556 (PMC3961385; doi:10.1371/journal.pone.0092556)
Supplement: Note S2 — Removal of data in regions of PCR allelic-bias. (DOCX) [file pone.0092556.s005.docx]

“Characterisation of genetic variation in *ST8SIA2* and its interaction region in NCAM1 in patients with bipolar disorder”

**AD Shaw, Y Tiwari, W Kaplan, A Heath, PB Mitchell, PR Schofield, JM Fullerton**

## Supplementary Note 2: Removal of data in regions of PCR allelic-bias

We examined a known source of inaccuracy within genotype calling from amplicon-based methodologies. Allelic bias, in which the product produced from one chromosome of a pair is more efficiently amplified by PCR despite equal amounts of input DNA from both chromosomes, can lead to false homozygous genotype calls for SNPs within the amplicon.

The allelic bias is often caused by heterozygous variation within primer binding sites. Although our primers were designed to avoid known human variants, many additional variants have been reported since the design stage, with the release of 1000 genomes data [1]. We found variation in the forward primer-binding site of three long-range amplicons (ST8-3, ST8-4 and ST8‑6), which affected a total of 12 individuals (4, 1 and 6 individuals per amplicon, respectively). By examining the balance between reads supporting either reference or variant alleles at well-covered sites, and examining genotype concordance at poorly-covered sites, we identified evidence of allele bias due to primer binding site variants (data not shown). We ran our haplotype phasing analysis after removal of sequencing data derived from individuals with heterozygous variation in primer binding sites (Figure 4).

**References**

1. The 1000 Genomes Project Consortium. (2010) A map of human genome variation from population-scale sequencing. Nature 467: 1061–1073. doi:10.1038/nature09534.
